# Geomorphology Drives Amphibian Beta Diversity in Atlantic Forest Lowlands of Southeastern Brazil

**DOI:** 10.1371/journal.pone.0153977

**Published:** 2016-05-12

**Authors:** Amom Mendes Luiz, Thiago Augusto Leão-Pires, Ricardo J. Sawaya

**Affiliations:** 1 Programa de Pós-Graduação em Ecologia, Instituto de Biologia, Universidade Estadual de Campinas (UNICAMP), CP 6109, 3083–970, Campinas, São Paulo, Brazil; 2 Setor de Ecologia e Biologia Evolutiva, Universidade Federal de São Paulo (UNIFESP), 09972–270, Diadema, São Paulo, Brazil; Trier University, GERMANY

## Abstract

Beta diversity patterns are the outcome of multiple processes operating at different scales. Amphibian assemblages seem to be affected by contemporary climate and dispersal-based processes. However, historical processes involved in present patterns of beta diversity remain poorly understood. We assess and disentangle geomorphological, climatic and spatial drivers of amphibian beta diversity in coastal lowlands of the Atlantic Forest, southeastern Brazil. We tested the hypothesis that geomorphological factors are more important in structuring anuran beta diversity than climatic and spatial factors. We obtained species composition via field survey (N = 766 individuals), museum specimens (N = 9,730) and literature records (N = 4,763). Sampling area was divided in four spatially explicit geomorphological units, representing historical predictors. Climatic descriptors were represented by the first two axis of a Principal Component Analysis. Spatial predictors in different spatial scales were described by Moran Eigenvector Maps. Redundancy Analysis was implemented to partition the explained variation of species composition by geomorphological, climatic and spatial predictors. Moreover, spatial autocorrelation analyses were used to test neutral theory predictions. Beta diversity was spatially structured in broader scales. Shared fraction between climatic and geomorphological variables was an important predictor of species composition (13%), as well as broad scale spatial predictors (13%). However, geomorphological variables alone were the most important predictor of beta diversity (42%). Historical factors related to geomorphology must have played a crucial role in structuring amphibian beta diversity. The complex relationships between geomorphological history and climatic gradients generated by the Serra do Mar Precambrian basements were also important. We highlight the importance of combining spatially explicit historical and contemporary predictors for understanding and disentangling major drivers of beta diversity patterns.

## Introduction

Spatial organization of diversity patterns is one of the most interesting properties of ecological communities [1]. The last two decades have witnessed a growing focus on study of spatial patterns of variation in species composition [2,3]. This variation was termed by Whittaker’s seminal papers [4,5] as the beta diversity component of species diversity. The particular interest in beta diversity stems from the fact that understanding the variation in species composition allows a better view on what set of processes drives biodiversity [6,7]. Simple species counts as alpha diversity could not express such explicit variation in species identity [8]. Moreover, beta diversity studies provide the so-called “mensurative experiments”, since broad-scale manipulative experiments are not feasible [9,1].

Considering the complex nature of ecological communities, any dichotomized perspective as “regional versus local” diversity of structuring processes would be oversimplistic [8,10–13]. However, such paradigmatic divisions can be useful as a starting point towards an integrative framework and to disentangle the relative importance of different processes influencing the structure of ecological communities (see [14–16]). Niche differentiation among co-occurring species has been frequently invoked as the primary process in structuring ecological communities, an idea deeply rooted in niche theory [17–19]. There are several important factors influencing diversity patterns under the umbrella of niche-based processes, such as use of limited resources, predation, parasitism and competition, as well as environmental conditions to which species are adapted [19]. One of the predictions of beta diversity patterns regarding niche-based processes is that species distributions and community structure are closely tied to environmental variables, which in turn are ecologically relevant to species niche [6,7]. This model has been termed as the environmental control [17,20–21].

However, processes occurring in larger spatio-temporal scales can also play important roles in structuring communities [22,23]. Processes occurring in regional scales, such as speciation and dispersal from the species pool, can be balanced by negative effects of competitive exclusion and unfavorable environmental conditions in local communities [23,24]. Geological history, for instance, could generate barriers and corridors, creating spatial patterns in species distributions and consequently in beta diversity [23,24]. Although geological events could be considered deterministic, as niche-based processes, it can be distinguished by representing larger spatio-temporal processes affecting species in an evolutionary scale [24]. Furthermore, this evolutionary imprint in species distributions can take precedence among processes that shape community structure and therefore can be more relevant to the current patterns of beta diversity.

The focus in processes operating in larger scales also highlighted how stochastic elements may be relevant in structuring diversity patterns [19,23]. Neutral theory makes clear assumptions about preeminence of random processes in structuring communities [11,25,26]. The neutral theory assumes individuals as ecologically equivalents and consequently considers that ecological drift is a major driver of community structure. Therefore, the variation in species composition would be the outcome of stochastic but spatially restricted dispersion [7], rather than niche differentiation among species. This neutral dynamics would generate clear positive spatial autocorrelation structures in ecological communities [27].

One of the most important challenges to ecologists is to understand the relative contributions of several processes at distinct spatial scales and their interactions in structuring ecological communities [8]. The partitioning approach using different sets of variables, such as climatic and geological data, allows to disentangle different processes influencing assemblage structure, and it has been a popular and useful approach in community ecology [28,29]. Spatial variables *per se* have been regarded as important surrogates to identify some of these potential multiscale processes underlying variation in species composition, rather than a nuisance of spatial dependence in statistical modeling [30–33].

A recent method has been proposed to understand if spatial structure of communities could be explained by neutral dynamics or non-measured spatially structured environmental variables (see [27]). If there is a high degree of non-neutrality in species abundance patterns, i.e., if species abundance is determined by similar ecological factors, we should expect a significant correlation among abundances within species groups and similar correlograms [27]. Therefore, incorporating spatial variables in models has helped to understand and disentangle the relative roles of niche, historical and neutral processes in community ecology [30,33].

Amphibians have peculiar traits, such as permeable skin and complex life histories, and their geographic distributions are known to be affected by abiotic factors, particularly temperature, precipitation and humidity [34–36]. These environmental conditions directly affect the physiological performance and in turn the distribution of anuran species [34]. It has been assumed that dispersal abilities of anurans are limited [37]. This suggests that both constraints to dispersal, such as geological barriers or neutral dynamics under restricted dispersal, could be relevant drivers of amphibian beta diversity.

We assess herein the geomorphological, climatic and spatial drivers of beta diversity patterns of anurans from the threatened coastal lowlands of Atlantic Forest, southeastern Brazil. Although multiple processes influence patterns of beta diversity, we test the hypothesis that geomorphological factors are more important in structuring anuran beta diversity than climatic and spatial factors. We have also implemented the analytical approach of Diniz-Filho *et al*. [27] to test the influence of neutral dynamics in spatial structure of anuran communities. We highlight the importance of combining spatially explicit historical and contemporary predictors for a better comprehension of drivers of beta diversity.

## Material and Methods

### Ethics statement

Collection permits were provided by Instituto Chico Mendes de Conservação da Biodiversidade (ICMBio) (#22805–1). Field studies did not involve endangered or protected species. We restricted manipulation of animals in the field to minimal as we sampled species by acoustic and visual searching [38] (see also below in Data sources, Species occurrence data). Less than ten individuals per species were captured in accordance to the collection permits, killed using lidocaine, and preserved in 70% alcohol as vouchers, following the suggestion of McDiarmid [39] for amphibians. In 2010, beginning of the field sampling planning, there was no need for approval by any Institutional Animal Care and Use Committee (IACUC) or equivalent animal ethics committee in Brazil and our graduation program. All sampling procedures were reviewed and specifically approved as part of obtaining the field permits by ICMBio (see above) and Comissão Técnico-Científica do Instituto Florestal (COTEC; a committee of Instituto Florestal, a public research agency and owner of the reserves) (Processo SMA # 260108–002.279/2010).

### Study area

**Fig 1:**
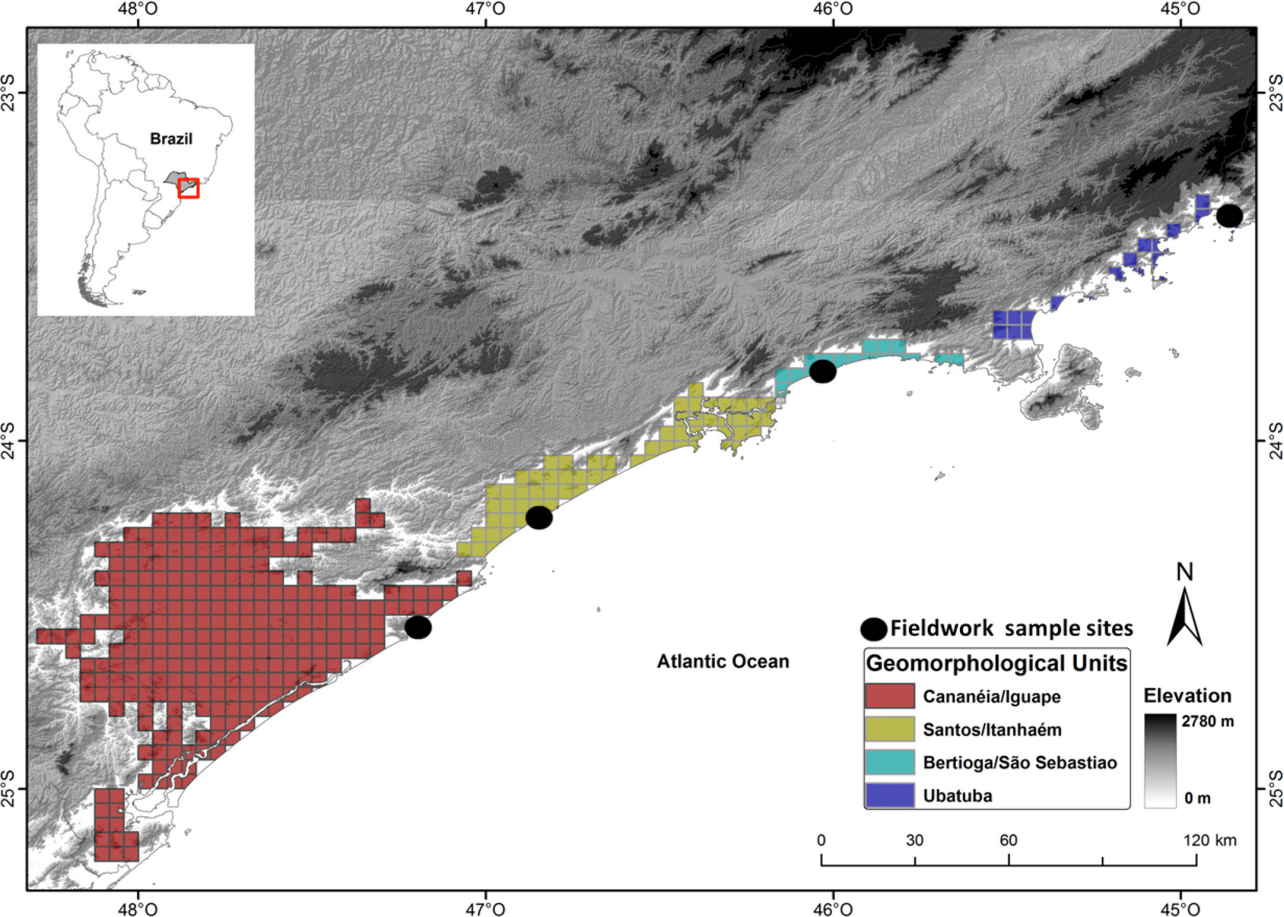
Study region on the coastal plains of Precambrian basements in Serra do Mar, São Paulo state, southeastern Brazil. The grid cells are grouped according to their respective geomorphological unit described by Suguio and Martin [40]. The main divisions group Bertioga/São Sebastião and Ubatuba units in north (green and purple cells, respectively) where Precambrian basements reach the sea, and Cananéia/Iguape and Santos/Itanhaém (red and yellow cells, respectively) units in south have larger coastal plains generated by differential uplift of the Serra do Mar. Points represent sample sites where we carried out fieldwork.

The study area encompasses the coastal region of São Paulo state, with about 550 km of extension (Fig 1). Its geomorphological history was described by Sugio and Martin [40]: the coastal plains of this region are bounded on southern and northern portions by Precambrian basements corresponding to the Serra do Mar range. The northern region is characterized by presence of Precambrian basements reaching the sea, creating relatively small coastal plains. The southern region shows wider sedimentary coastal plains due to differential uplift of the Precambrian basements (Fig 1). The coastal plains within this region are naturally separated by narrow headlands of Precambrian rocks, splitting the region in four geomorphological subdivisions (Fig 1). It is worth noting that these Precambrian natural divisions are much older than the coastal plains where ecological communities have established after Quaternary marine regressions [40]. These four geomorphological units were used here as historical predictors of species composition in the multivariate analysis (see below).

The vegetation is mainly composed by *restinga* forests and ombrophilous lowland forests [41]. Bromeliads (Bromeliaceae) are among the most important families of *restinga* vegetation [42]. Some Bromeliads are considered nurse plants, which favor the establishment of trees and shrubs species [43] and provide shelter and reproductive resource for anurans. The occurrence of endangered species such as *Euterpe edulis* [42], as well as a rich and structured amphibian fauna (see [44]), makes this ecosystem a priority area for conservation [45].

### Data sources

#### Species occurrence data

We carried out fieldwork at four sites:, Ubatuba (23°21'27"S; 44°50'51"W), Bertioga/São Sebastião (23°45'24"S; 45°55'29"W), Santos/Itanhaém (24°12'00"S; 46°54'46"W) and Cananéia/Iguape (24°36'48"S; 47°21'04"W). Each of them is located in one of the four geomorphological subdivisions (Fig 1). We selected five water bodies for “search in reproductive sites” [37], including temporary and permanent ponds in each site, totaling 20 sampled ponds. We selected five transects within forests for the “complete species inventory” [37], at least 500 m apart from each other in each site, totaling 20 sampled transects. Species were sampled by acoustic records and searching on all suitable microhabitats along each transect and pond. Each site was sampled in three occasions from October 2011 to May 2012, in order to maximize the probability of finding frogs during most of the breeding season. We recorded 766 specimens belonging to 40 species combining all sampling field methods (see S1 Table).

We also examined 9,730 specimens belonging to 57 species with known records for the study area deposited in the scientific collections Célio F. B. Haddad (CFBH), from Universidade Estadual Paulista, Campus Rio Claro, and Museu de Zoologia da Universidade Estadual de Campinas Adão José Cardoso (ZUEC; see locality records in S2 Table). We excluded rheophilic species (genera *Cycloramphus*, *Crossodactylus*, *Hylodes* and *Thoropa*) as such species are associated with rocky streams in foothill and mountain environments in boundaries of *restingas* of the study region, and because such species are typically associated with ombrophilous forests. We complemented our geographic distribution database with literature data (N = 4,763 specimens; S1 Table). Through all data sources, *i*.*e*., fieldwork, museum and literature data, we recorded 57 species in the study region.

We described geographic distributions by means of α-hull polygons, using *alphahull* R package [46]. This method calculates the average length of all lines connecting specimens records through a Delaunay triangulation, and excludes lines longer than this average length, avoiding biases of the minimum polygon convex method related to shape and species habitat [47]. This method was applied because the polygons can represent distributions continuously, which is more consistent with study area range (~ 500 km), and since species recorded do not show known disjunct populations in the study area. The polygons of species distributions were produced to record species presence in a 2.5’ grid cell system (~ 25 km²) up to 100 m above sea level (Fig 1), using functions of R package *raster* [48]. As the southernmost subdivision has disproportionally more cells than other geomorphological units (see Fig 1), we randomly sampled 60 cells of this unit, and the final data set (**Y**) included 164 rows of sites and 57 columns of species, Hellinger transformed [29].

We used the raw-data approach to assess beta diversity [7], using a presence-absence matrix Hellinger transformed. The concept of beta diversity is contentious along the literature [6,7]. Thus we choose herein the raw-data approach (*sensu* [7]) because our questions have focused the community composition variation instead of variation in beta diversity along space (see discussion in [6,7]).

#### Climatic and geomorphological variables

Climatic descriptors were compiled from 19 bioclimatic variables of the WorldClim database [49] in a 2.5’ resolution. We extracted values of climatic variables from each cell in our grid generating a matrix of 164 rows representing sites and 19 columns representing climatic variables. We excluded correlated variables (Pearson’s r > 0.85) and those which we do not have a relevant and/or plausible interpretation about their influence on anuran biology and distribution. Thus, only four variables were selected and used on subsequent analyses, namely: temperature seasonality, mean temperature of the coldest month, precipitation seasonality and precipitation of the driest quarter [50]. We then performed a Principal Component Analysis (PCA) based on a correlation matrix in order to avoid multicollinearity among remaining climatic variables, which artificially increases explained variation and consequently causes type I error [29]. Based on eigenvalues and consequently on the proportion explained by each PCA axis, we selected the first two axes, accounting for 67.4% and 24.4% of variance, respectively (Table 1). These first two axes were used as the matrix of our climatic predictors (**Clim**) (see S1 Text).

**Table 1:** Loadings from the Principal Component Analysis for four climatic variables and 164 combined cells used in the account.

Climatic Variables	PC 1	PC 2
**Temperature seasonality**	0.55	-0.30
**Precipitation of the driest quarter**	-0.53	-0.39
**Precipitation seasonality**	0.49	0.52
**Mean temperature of coldest month**	-0.42	0.69
**Variation accounted**	67.4%	24.4%

We used the four geomorphological units defined by Suguio and Martin [40] as a categorical predictor of species composition, in which each cell pertains to one of the four units: Cananéia-Iguape, Itanhaém-Santos, Bertioga-São Sebastião and Ubatuba (Fig 1). The categorical variables (**Geo**) were coded as dummy variables [51]. It is worth noting that these geomorphological units are spatially explicit, however they have a different biological meaning when compared to the purely spatial component assessed herein. These units can represent historical biogeographic processes influencing beta diversity in the region (see [52]).

### Analyses

Spatial linear trends were removed in order to model detailed spatial structures, through a detrending procedure on species composition matrix (see [29,51] and S1 Text). We then generated spatial predictors to explain beta diversity by implementing a spatial eigenfunction analysis [29,31] based on Moran Eigenvector Maps (MEMs; [32,53]). This method consists on eigenvector decomposition of connectivity matrices. The eigenvalues of MEMs represent Moran’s I statistic and describe spatial autocorrelation in different spatial scales (see details in [32]).

A crucial step in MEM approach is the definition of a neighborhood matrix, which describes spatial relationship among objects [32]. In our case, it was necessary to define which sites (i.e., cells) are neighbors and which are not. We used a heuristic approach to define neighborhood relationship between sites in which we tested several neighborhood distances, beginning with distance obtained through minimum spanning tree algorithm up to a maximum distance between sites [51]. The best distance to construct the neighborhood matrix was selected based on corrected Akaike information criteria using *test*.*W* function of *spacemakeR* package [51,54]. MEMs with positive eigenvalues were generated from the best neighborhood matrix, and the best set of MEMs was selected through forward selection with double criteria based on adjusted R² statistics [29]. Positive eigenvalues represent positive spatial autocorrelation, because they are linearly correlated with Moran’s I index [32].

Fifty neighborhood matrices were tested with maximum distances to the nearest cell varying from 22.4 (obtained by minimum spanning tree algorithm) to 380.0 km, which is the maximum distance between cells in our grid. Model selection based on corrected Akaike information criteria (AICc) of distinct neighbourhood matrices had AICc values ranging from -50.2 to 167.2. The best neighborhood matrix model (AICc = -50.2) has a maximum distance to nearest neighbor of 22.4 km. Forward selection in MEMs with positive eigenvalues resulted in a set of spatial variables with 22 MEMs (see details in S1 Text).

We divided the MEMs in submodels according to the scales they represent in order to describe spatial variation in species composition in distinct scales. This is possible owing to the orthogonal property of MEMs and multiscale spatial structure they are able to model. The submodels and their associated scales can be defined according to (i) the study aims, (ii) the similarity in the periodicity of spatial structure of significant MEMs [55,56], and (iii) by visual inspection of eigenvectors in the map [57]. We defined two submodels representing broad (**Broad**) and fine (**Fine**) scales used as spatial predictors in the multivariate analysis (see examples in S1 Fig). These two scales can represent ecological processes spatially structured at different scales, such as climatic conditions, dispersal and interspecific interactions. Then, we grouped MEMs in broad and fine scales after visual inspection on the map and similarity in periodicity. Ten MEMs were classified as broad scale and 12 as fine scale (S1 Text).

Considering the spatial nature of geomorphological variables, correlations among some MEMs and geomorphological units would be expected, as such MEMs are mathematical representations of spatial structure of geomorphological units. Therefore, we removed MEMs correlated with geomorphological variables (Pearson’s r > 0.3) to avoid collinearity in the model. We excluded five broad scale MEMs (MEMs 2, 3, 4, 6 and 10) as they showed correlation with geomorphological units greater than r > 0.3, and could lead to misinterpretation of spatial structure of beta diversity. Additionally, adopting this procedure, we improve the interpretation of potential explanation by spatial fraction, in the light of geomorphological history.

We implemented a redundancy analysis (RDA) and partial RDA to partition the explained variation in species composition, attributed to different sets of explanatory variables: climatic, geomorphological, broad and fine scale predictors [28,53]. The explained variation was expressed by unbiased adjusted R² statistics (R²_adj_, see [58]). We used a hierarchical approach to partition the explained variation, which assumes a downscale priority of processes structuring the species composition, such as geomorphological history and broad scale climatic variables (see [56]). Total variation explained by climate, geomorphological units, broad and fine scales were decomposed in 14 fractions corresponding to individual and shared fractions of predictors. Redundancy analysis was preferred because previous detrended correspondence analysis revealed a short gradient in the response matrix (see [56]). All procedures were performed using functions of *vegan* R package [57]. The significance of independent fractions was tested by permutation through *anova*.*cca* function of *vegan* package [57].

To assess if the fraction explained by spatial variables reflects the influence of neutral dynamics or the effects of non-measured environmental variables, we used the protocol proposed by Diniz-Filho *et al*. [27]. This procedure is based on models of genetic differentiation among populations in geographic space and tests explicitly the predictions of neutral theory: (i) species abundances are independent of each other; (ii) species abundances have similar correlogram shapes and spatial autocorrelation magnitudes; and (iii) non-neutrality driving species abundances creates correlation patterns among species abundances with similar correlograms. To test these predictions, we constructed Moran’s I correlograms and produced a matrix of pairwise Manhattan distances among species correlograms (**M**). A second matrix of pairwise correlations among species incidences were also constructed (**R**). Under neutral dynamics, we expect no correlation between constructed matrices of pairwise distances among species correlograms (**M**) and pairwise correlation among species profiles (**R**). As we assessed only the fraction explained by spatial variables, all values in the matrices used to construct **R** and **M** are predicted by spatial variables extracted with *predict* function in *vegan* R package [59]. Although this method has been originally developed to abundance data, there would be no problem to use binary data in this approach (J. A. F. Diniz-Filho, *pers*. *comm*.). Moreover, Moran’s I correlograms is a robust approach for presence-absence data (see [60]) and recent simulations have shown that presence/absence and abundance data are complementary in community composition analyses [61].

## Results

We recorded 57 amphibian anuran species belonging to eight families, being 40 recorded in field sampling (see S1 Table). Occurrence of species in each geomorphological unit were unequal, with 12 species occurring in one unit, three in two units, six in three units, and 36 widespread throughout of study region (S2 Table).

**Fig 2:**
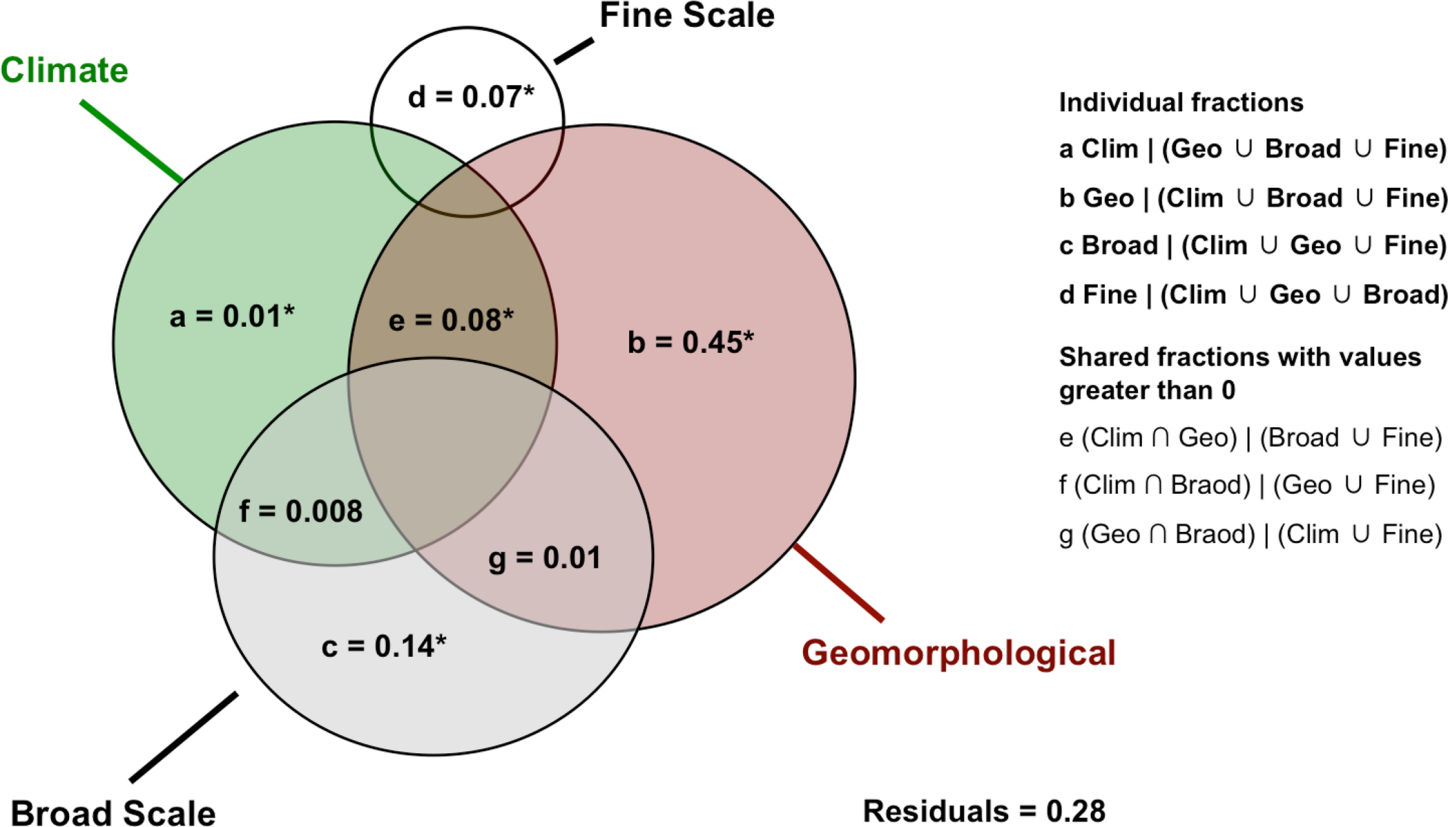
Variation in partitioning analysis. Explained variation of amphibian anuran species composition was partitioned in shared and unique fractions of climatic (**Clim**), geomorphological (**Geo**), broad (**Broad**) and fine (**Fine**) scale predictors. Fractions a, b, c and d represent the unique effects of **Clim**, **Geo**, **Broad** and **Fine**, respectively. Fractions e to g represent intersections, or joint effects of different predictors after controlling for effects of remaining predictors (i.e., “covariables” in RDA model). Residuals are the fraction not explained by either predictors included in the model. The upright and downright boxes represent the notations to each set (i.e., fraction). The symbol “**∩**” represent intersection, “∪” represent union, and “|**”** represent after controlling for. Fractions with values lower than 0 are not showed in the diagram. (*) means significant fractions after permutations tests (*P* < 0.05).

All individual fractions of RDA analyses were significant at alpha = 0.05. The whole model with climatic, geomorphological, broad and fine scales described 73% of the beta diversity in the study region (Fig 2). Geomorphological history alone was the most important fraction, describing 42% of variation in beta diversity (fraction b; Fig 2). Both, fraction shared by geomorphological and climatic variables (fraction b; Fig 2) and spatial variables classified as broad scales described 13% of variation. Spatial variables grouped in fine scales described 8% of beta diversity (fraction d), and variation explained only by climatic predictors was 2% (fractions a).

**Fig 3:**
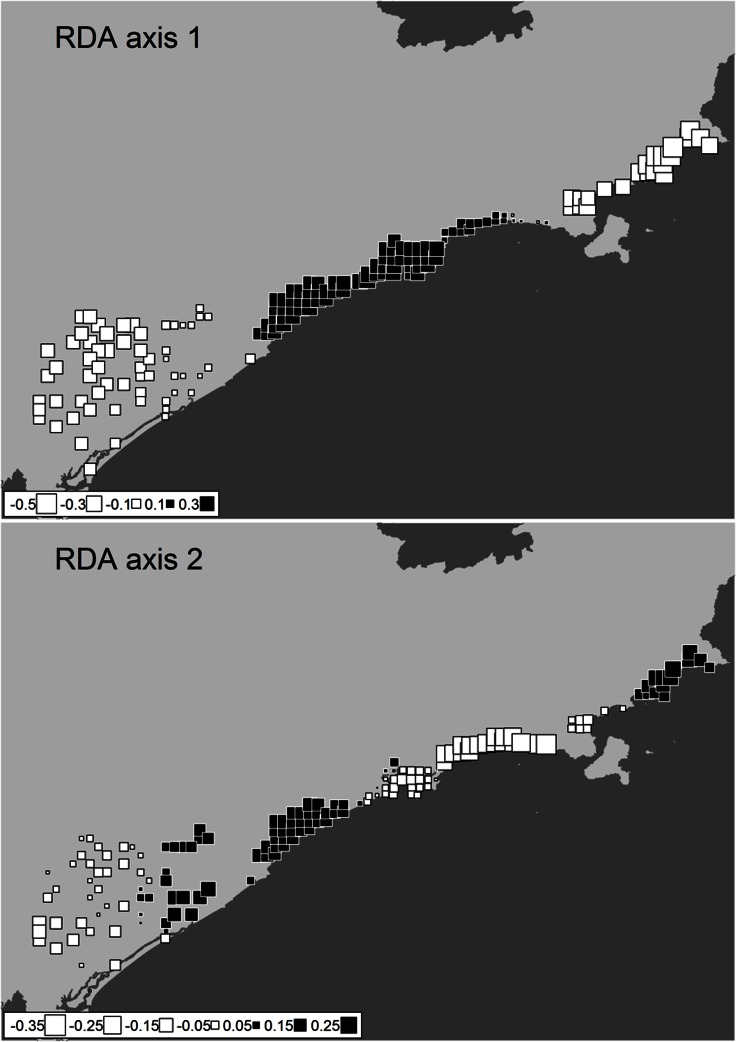
Study region with site scores of the two first axes of partial redundancy analysis (RDA). Scores of the two first axes of partial redundancy analysis were constrained by all predictors: geomorphological, climatic, broad, and fine scale variables. The cell size represents scores of axes and color represents negative and positive scores (white and black, respectively). The first axis (a) described 55% and the second (b) described 14.2% of variation of anuran composition along Atlantic Forest lowlands in coastal plains of southeastern Brazil.

The two first RDA axes mapped in studied area represented the ordination of beta diversity constrained by all predictors, and accounted for 55% and 14.2% of variation, respectively (Fig 3). Beta diversity is spatially structured in relation to all predictors, but mainly by geomorphological variables, the most important predictor for variation in species composition of coastal plains.

We used the whole spatial fraction that represents the variation explained by broad and fine spatial variables, and described 21% of beta diversity, to evaluate the influence of neutral dynamics in this fraction. Average means of Manhattan distances between species correlograms (**M**) was 4.18 (± 2.1 SD). The correlation among species profiles (**R**) was low (mean = 0.11 ± 0.5 SD). Mantel correlation between **M** and **R** was -0.69 (*P* = 1.0).

## Discussion

We found a clear spatial structure of beta diversity throughout the Atlantic Forest coastal plains of the São Paulo state, in southeastern Brazil. Considering the spatial context of our study area, we found that spatial pattern of beta diversity is structured mostly in broader scales. This can be evidenced by the map with scores of first two RDA axes (Fig 3), and by predictors that explained most part of variation in species composition (Fig 2): the geomorphological history which is itself a spatial predictor of broader scales, and the MEMs classified as broad scale (e.g., MEMs 1 and 2 in S1 Fig). The reasoning behind this finding is that ecological systems could be affected by processes following a downscale spatial hierarchy [56]. We can thus expect that processes occurring in broad scales such as geomorphology could generate broad-scale signatures in structure of ecological communities [56] as our results evidenced herein.

The fraction explained only by climatic variables was low (2%). However, the fraction shared by climate and geomorphological units explained 13% of total variation. Shared fractions between climatic or other environmental variables, and spatially explicit variables such as geomorphological units or MEMs, are difficult to interpret because they could represent ambiguous sources of variation [53]. There are some potential explanations for the source of variation in these shared fractions: (i) if these fractions represent spatial autocorrelations in the community composition owing to measured climatic factors spatially structured, they can be interpreted as spatial structure affected by climate; or (ii) if there are relevant missing predictors which are spatially structured and such predictors covariate with measured climatic variables, then this fraction could not be interpreted unambiguously (see [53]). In fact, Warren *et al*. [13] addressed similar issues, considering the autocorrelation of environmental variables and how it may influence the geography of species diversification.

There would be a third possibility to interpret the fraction shared by climatic variables and geomorphological history regarding our study region. Coastal plains in the study region are surrounded by a Precambrian basement, the Serra do Mar range, which defines the geomorphology of the region. The Serra do Mar influences the climate of the region retaining wet air masses, causing orographic rains and distinct climates throughout the geomorphological units [41]. For instance, southernmost units are in a colder region because of its geographic position and their coastal plains are more distant from Serra do Mar slopes (see Fig 1). Consequently, this unit shows different climate with greater seasonality and lower precipitation (pers. obs.). Then, it is reasonable to assume that the fraction shared by geomorphological history and climatic variables represents a synergic influence of these predictors to drive the spatial structure of beta diversity. This would be expected since the well-known influence of climatic variables shaping species distributions, which affects the species niches (see [62]) and consequently the structure of ecological communities. Temperature, for instance, directly affects anuran diversity patterns seasonality by constraining the distribution of species with narrow niches to occur in environments with more stable climates [63].

Spatial variables structured in broad scales (13%; Fig 2) were another relevant predictor of variation in species composition. Moreover, fine scale spatial variables explained significantly 8% of the beta diversity. It is important to note that these spatial fractions cannot be interpreted unambiguously, because they may reflect the effect of spatially structured unmeasured environmental variables, as well as neutral processes generating positive spatial autocorrelation in ecological communities [27]. Our results obtained through spatial autocorrelation analysis show that such pure spatial fractions are not congruent with what would be expected by neutral dynamics. Correlograms showed high mean distance, indicating that species have not similar spatial autocorrelation patterns, which in turn would be a major assumption of neutral theory [27]. Additionally, the relationship between Manhattan distances (M) and incidence correlations (R) was negative, evidencing that species with similar correlograms have high incidence correlations. These findings indicate that beta diversity patterns explained by pure spatial predictors could in fact, represent the effect unmeasured environmental variables, rather than neutral dynamics. Further studies should compare incidence and abundance data with simulated datasets, but such analysis are beyond the scope of the present work.

Geomorphological history stood out as the most important driver of variation in anuran species composition in the studied area (42%; Fig 2). This result indicates that historical processes are more important than contemporary processes, such as specific local climatic conditions, in shaping community assembly and regional beta diversity patterns. There are some theoretical and empirical reasons to ground this finding. First, current patterns of diversity could be the outcome of historical processes occurring in regional scales [22]. Geomorphological barriers, for instance, can constrain the dispersal of species among areas [22] and even relatively smaller barriers could produce spatial patterns of species distribution and composition among areas, considering limited dispersal ability of anurans [39]. The coastal plains of study region were shaped during the Quaternary period through deposition of sediments after repeated oceanic incursions [40]. However, these sediments were deposited on older Pre-Cambrian rocks [40], which defined the geological scenario where coastal plains were posteriorly shaped. Afterwards, current anuran communities were established through colonization from surrounding habitats, such as ombrophilous forests of the Serra do Mar. This geological scenario divides the region through narrow headlands with higher elevations (Fig 1), creating potential barriers to species for dispersal among geomorphological units. This could evidence that the complex geomorphological history of the region has played an important role in spatial variation of anuran species composition.

This historical influence in beta diversity patterns of the region could indicate the effect of other historical processes rather than exclusively potential barriers to dispersal. Beta diversity patterns can be related to diversification patterns of regional biotas [64]. Among hypotheses of diversification patterns in the Atlantic Forest, the most well-known is based on the Pleistocene refugia model [65–68]. This hypothesis posits that Pleistocene glacial cycles have caused multiple vicariant events in populations of species not adapted to non-forested habitats, owing to the retraction of suitable forested patches, and creating stable refugia during the dramatic climatic changes in glacial periods [65,66]. An alternative hypothesis emphasizes older processes like orogeny in the late Tertiary, characterized by uplifting of the east coast of Brazil with further geographic and climatic modifications [66].

Phylogeographic studies have shown mixed species-specific results, supporting these two presumed mechanisms on diversification in the Atlantic Forest (e.g., [52,66,68,69]). However, such studies have revealed ubiquitous spatial patterns of divergence, and splitting lineages in southern and northern regions of São Paulo state (see [52,66,69,70]). These patterns could be due to potential refugia in northern and southern regions [69] and/or by recent southern colonization by populations from northern portions of the region during the Pleistocene [70], or even by phylogeographic breaks associated with older tectonic activity [52]. Moreover, lowland restricted species are presumably unable to tolerate cold temperatures of montane regions, and should be more susceptible to dramatic climatic oscillations of the Pleistocene (see [52]). It is plausible that such events related to patterns of divergence among lineages have also influenced patterns of species distributions and could consequently generate a spatial signature in beta diversity patterns along the coastal plains of the study region. Thus, although the known limitation in using surrogates for species isolation, such as geographical barriers or distance, geomorphological history itself may be a suitable approach in the attempt to include effects of speciation history in beta diversity studies, since speciation can be result of vicariance caused by geological events [44].

Studies on Atlantic Forest amphibians using broader scales and distinct facets of biodiversity, such as biogeographic patterns [71] and phylogenetic diversity [36], have shown both similar and distinct results from our study. For instance, Vasconcelos *et al*. [71] found high congruence among ecoregions and anuran biogeographic patterns along Atlantic Forest, and revealed a relatively homogeneous fauna in southeastern Brazil. Although our results could not be directly compared to that study, owing to distinct spatial scales, Vasconcelos *et al*. [71] proposed that southeastern Brazil with its rich anuran fauna represents a distinct biogeographic region, characterized by small-ranged species isolated by mountain chains such as the Serra do Mar range. Our results provide similar empirical evidence, as beta diversity patterns were strongly influenced by geomorphological divisions of Pre-Cambrian basements forming the Serra do Mar mountain range. Although southeastern Brazil has been classified as relatively homogeneous by Vasconcelos *et al*. [71], it is not surprising that if we move to lower scales we find compositional heterogeneity and higher beta diversity. Indeed, our scale (about ~25 km²) is two orders of magnitude lower than that of Vasconcelos *et al*. [71] (about ~2500 km²). Such higher beta diversity at smaller scales is related to small-ranged species, as quoted by Vasconcelos *et al*. [71], which in turn are strongly influenced by geomorphological history.

As correlative approaches do not imply causation processes, attention is necessary to interpret fractions of explained variation [2]. Additionally, recent criticisms have pointed out statistical limitations of variation partitioning approach to estimate accurately the relative contributions of predictors (e.g., [72]). However, this approach still provides a first step to disentangle important processes related to beta diversity patterns (e.g., [33,73]) as shown here. Our results evidence a clear influence of historical processes shaping the spatial structure of anuran beta diversity in coastal plains of the Atlantic forest. This historical influence can shed light on processes related to the origin and maintenance of biodiversity in community level in the Atlantic Forest hotspot. Nonetheless, our study also highlights the need to consider spatially explicit information on both historical and contemporary variables, in order to evidence the synergic effects of distinct sets of predictors to beta diversity, as such effects are inherent from the complex nature of ecological communities.

## Supporting Information

S1 Table Localities of species occurrences. Localities of species occurrences based on fieldwork, scientific collections and literature data.
(DOC)

S2 Table Species occurrences in the four geomorphological units of the study region. Ampibian anuran species recorded in *restinga* forests from coastal plains of São Paulo state and its occurrence in four geomorphological units defined by Suguio and Martin ([40]; see Methods and Fig 1): Cananéia/Iguape, Itanhaém/Santos, Bertioga/São Sebastião and Ubatuba.
(DOC)

S1 Text Analytical roadmap. Commented R Codes and link to data sources of all analyses.
(PDF)

S1 Fig Examples of Moran Eigenvector Maps classified as broad and fine scales. Examples of the Moran Eigenvector Maps (MEMs) classified as broad (i.e., MEM 1 and 2) and fine scales (i.e., MEM 18 and 20), which were used as spatial predictors of the variation of amphibian anuran composition. Squares in the maps represent scores of each site in MEMs. White squares have negative scores and black squares have positive scores. Squares sizes are proportional to score values. These values are also represented in the graphs below each map, allowing to identify similarity in periods (“modulation”) among MEMs.
(DOC)
