# Supplementary material for: Geomorphology Drives Amphibian Beta Diversity in Atlantic Forest Lowlands of Southeastern Brazil
Source: PLoS One. 2016 May 12;11(5):e0153977. doi: 10.1371/journal.pone.0153977 (PMC4865194; doi:10.1371/journal.pone.0153977)
Supplement: S1 Table — Localities of species occurrences based on fieldwork, scientific collections and literature data. (DOC) [file pone.0153977.s001.doc]

**Supporting Information**

**Geomorphology drives amphibian beta diversity**

**in Atlantic Forest lowlands of southeastern Brazil**

Amom Mendes Luiz, Thiago Augusto Leão-Pires & Ricardo J. Sawaya

**S1 Localities of species occurrences**

| **Species** | **State: Locality^1^** |
| --- | --- |
| **Brachycephalidae** |  |
| *Brachycephalus hermogenesi* | PR: São José dos Pinhais^sc^; SP: Cotia^2^, Juquitiba^2^, Peruíbe^2^, Piedade^sc^, Ribeirão Grande^2^, Salesópolis^2^, Tapiraí^sc^, Ubatuba^sc^ |
| *Ischnocnema bolbodactyla* | RJ: Angra dos Reis^3^, Parati^4^; SP: Caraguatatuba^sc^, Natividade da Serra^4^, Ubatuba^3,4^ |
| *Ischnocnema guentheri* | ES: Domingos Martins^sc^, Santa Teresa^sc,6^, Vargem Alta^sc^; MG: Belmiro Braga^7^, Camanducaia^sc^, Itamonte^sc^, Lima Duarte^sc^, Mariana^6^, Monte Verde^7^, São Roque de Minas^sc^, Viçosa^sc^; PR: Guaratuba^sc^, Morretes^sc,6^, Pinhão^7^, Piraraquara^6^, Volta Grande^6^; RJ: Angra dos Reis^7^, Ilha Grande^6^, Itatiaia^sc,6^, Leopoldina^6^, Nova Friburgo^sc^, , Parati ^sc,6^, Petrópolis ^sc^, Rio de Janeiro ^sc,7^, Santa Maria Madalena^sc^, Teresópolis^sc^, Valença^7^; RS: Cambará do Sul^6^, Canela^6^, São Francisco de Paula^6^; SC: Águas Mornas^6^, Blumenau^sc,7^, Campo Alegre^6^, Corupá^6^, Guaramirim^sc^, Ibirama^6^, Ilhota^7^, Lauro Müller^sc^, Joinville^7^, Novo Horizonte^6^, São Bento do Sul^sc,6^, Treviso^sc^; SP: Apiaí^sc^, Arapeí^sc^, Arujá^sc^, Atibaia^sc^, Biritiba-Mirim^sc^, Campos do Jordão^sc,6^, Cananéia^sc^, Caraguatatuba^6^, Cotia^sc^, Cubatão^sc,6^, Cunha^sc^, Eldorado^sc^, Guapiara^sc^, Iguape^sc^, Ilha Bela^sc,7^, Iporanga^sc^, Itanhaém^6^, Itapecirica da Serra^6^, Jundiaí^sc^, Juquitiba^sc^, Mauá^sc^, Mogi das Cruzes^sc^, Natividade da Serra^sc^, Peruíbe^sc,7^, Piedade^6^, Pilar do Sul^sc^, Piquete^6^, Praia Grande^sc^, Ribeirão Branco^sc^, Salesópolis^sc,6^, Santa Isabel^sc^, Santana de Parnaíba^sc^, Santo André^sc^, Santo Antonio do Pinhal^sc,6^, São Bernardo do Campo^sc^, São José do Barreiro^sc,7^, São Luís do Paraitinga^sc^, São Paulo^sc^, São Sebastião^sc^, Sete Barras^sc^, Tapiraí^sc^, Ubatuba^sc^ |
| *Ischnocnema parva* | RJ: Nova Friburgo^9^, Petrópolis^sc,8^, Santa Maria Madalena^sc^, Teresópolis^sc^; SP: Apiaí^sc^, Arujá^sc^, Atibaia^sc^, Biritiba-Mirim^sc^, Caraguatatuba^sc^, Cunha^sc^, Guarujá^sc^, Iguape*, Ilha Bela^sc^, Iporanga^sc^, Itanhaém^sc^,*, Jundiaí^sc^, Mairiporã^sc^, Mauá^sc^, Mogi das Cruzes^sc^, Natividade da Serra^sc^, Peruíbe*, Piedade^sc^, Ribeirão Grande^sc^, Salesópolis^sc^, Santo André^sc^, Santos^sc^, São Bernardo do Campo^sc^, São Luís do Paraitinga^sc^, São Paulo^sc^, Ubatuba^sc^ |
| **Bufonidae** |  |
| *Dendrophryniscus brevipollicatus* | RJ: Resende^sc^, Rio de Janeiro^sc,10,11^, Teresópolis^sc,11^; SP: Bertioga^sc^, Caraguatatuba^sc^, Cotia^sc,11^, Iguape ^sc,11^, Ilha Bela ^sc^, Itanhaém*^,sc^, Juquitiba ^sc,11^, Peruíbe^11^, Piedade ^sc^, Pilar do Sul ^sc^, Salesópolis ^sc,11^, Santo André ^sc,11^, Santos ^sc^, São Bernardo do Campo ^sc,11^, São Luís do Paraitinga ^sc,11^, São Paulo ^sc^, São Sebastião ^sc^, São Vicente ^sc^, Tapiraí ^sc^, Ubatuba*^,sc^ |
| *Dendrophryniscus leucomystax* | PR: Guaraqueçaba^11^, Morretes^sc,11^; RJ: Ilha Grande^11^, Nova Iguaçu^sc,10,11^; SP: Bertioga*, Cananéia^sc,11^, Caraguatatuba^sc^, Iguape^sc^, Ilha Comprida^sc^, Ilha do Cardoso^sc^, Itanhaém*^,sc^, Pariquera-Açú^sc^, Peruíbe*^,sc,11^, Registro^11^, Santo André^sc^, São Paulo^sc^, Ubatuba^sc^ |
| *Rhinella hoogmoedi* | AL: Murici^12^; BA: Arataca^12^, Camamu^12^, Canavieiras^12^, Ilhéus^sc,12^, Itabuna^12^, Itapebi^12^, Mata de São João^sc^, Nilo Peçanha^12^, Porto Seguro^12^, Prado^12^, Santa Cruz Cabrália ^sc,12^, São José da Vitória^12^, Una ^sc,12^, Uruçuca ^sc,12^, Valença^12^; CE: Guaramiranga^sc^, Pacoti^12^, Redenção^12^; ES: Linhares^sc^, Rio Doce^12^, Sooretama^12^; PR: Guaraqueçaba ^sc,12^, Morretes^sc^; PE: Água Preta^12^, Rio Formoso^12^; RJ: Angra dos Reis^12^, Duque de Caxias^12^, Magé^sc^, Mangaratiba^12^, Nova Iguaçu^12^, Rio das Ostras^12^, Rio de Janeiro^12^; SP: Araçatuba^12^, Bertioga*^,sc,12^, Cananéia^12^, Capão Bonito^12^, Caraguatatuba^12^, Conceição da Barra^sc^, Cubatão^12^, Eldorado^sc^, Guarujá^12^, Iguape*^,sc,12^, Itanhaém*, Juquiá^12^, Pariquera-Açú^12^, Peruíbe*^,sc,12^, Santos ^sc,12^, São Sebastião*^,sc,12^, São Vicente^12^, Sete Barras ^sc,12^ |
| *Rhinella icterica* | MG: Camanducaia^sc^, Itamonte^sc^; PR: Campina Grande do Sul^sc^, Cruz Machado^sc^, Morretes^sc^, Ortigueira^sc^, Quatro Barras^sc^, Tijucas do Sul^sc^; RJ: Cachoeiras de Macacu^sc^, Campos dos Goytacazes^sc^, Itatiaia^sc^, Nova Friburgo^sc^, Petrópolis^sc^, Santa Maria Madalena^sc^; RS: Bento Gonçalves^sc^, Cambará do Sul^sc^. Campo Bom, Derrubadas^sc^, Nova Roma do Sul^sc^, Sapiranga^sc^; SC: Blumenau^sc^, Bom Jardim da Serra^sc^, Botuverá^sc^, Corupá^sc^, Lauro Müller^sc^, Rio dos Cedros^sc^, São Bento do Sul^sc^, São Bonifácio^sc^, Treviso^sc^, Xavantina^sc^, Xanxerê^sc^; SP: Apiaí^sc^, Atibaia^sc^, Barra do Turvo^sc^, Campinas^sc^, Capão Bonito^sc^, Carapicuíba^13^, Cunha^sc^, Guapiara^sc^, Ibiúna^sc^, Iguape^sc^, Iporanga^sc^, Itapecerica da Serra^sc^, Jundiaí^sc^, Louveira^sc^, Mauá^sc^, Mogi das Cruzes^sc^, Peruíbe^sc^, Piedade^sc^, Pilar do Sul^sc^, Ribeirão Branco^sc^, Ribeirão Grande^sc^, Salesópolis^sc^, Santa Branca^sc^, Santo André^sc^, Santos^sc^, São José do Barreiro^sc^, São Luís do Paraitinga^sc^, São Paulo^sc^, São Sebastião^sc^, Suzano^sc^, Ubatuba^sc^ |
| *Rhinella ornata* | ARGENTINA: Corrientes^14^, Misiones^14^; BRAZIL: PR: Tibagi^sc^; RJ: Angra dos Reis^14^, Araruama^14^, Barra do Piraí^14^, Bom Jesus do Itabapoana^14^, Cachoeiras de Macacu^14^, Cambuci^14^, Duque de Caxias^14^, Engenheiro Paulo de Frontin^14^, Ilha Grande^14^, Itaguaí^14^, Itatiaia^14^, Macaé^14^, Mangaratiba^14^, Maricá^14^, Mendes^14^, Miguel Pereira^14^, Niterói^14^, Nova Friburgo^14^, Nova Iguaçu^14^, Paraíba do Sul^14^, Parati^sc,14^, Petrópolis ^sc,14^, Resende^14^, Rio Bonito^14^, Rio de Janeiro^14^, Santa Maria Madalena^14^, Silva Jardim^14^, Teresópolis^14^, Três Rios^14^; RS: Derrubadas^sc^; SP: Angatuba^sc^, Apiaí ^sc,14^, Arapeí^sc^, Artur Nogueira^sc^, Arujá^sc^, Atibaia^sc^, Bananal^14^, Barra do Turvo^sc^, Barueri^14^, Bauru^14^, Bertioga*, Biritiba-Mirim^sc^, Botucatu^14^, Brotas^sc^, Buri^sc^, Cabreúva^sc^, Caieiras ^sc,14^, Campinas ^sc,14^, Campos do Jordão^14^, Capão Bonito^sc^, Caraguatatuba ^sc,14^, Cubatão^14^, Eldorado^14^, Embu^14^, Ferraz de Vasconcelos^14^, Franca^14^, Garça^14^, Guareí^sc^, Guarujá ^sc,14^, Guarulhos^14^, Iguape*^,sc,14^, Ilha Bela ^sc,14^, Ilha Comprida^sc^, Iporanga ^sc,14^, Itaberá^14^, Itanhaém*^,14^, Itapecerica da Serra^14^, Itapevi^14^, Itariri^14^, Itatiba, Itu^14^, Jacupiranga^14^, Jundiaí^sc^, Juquiá^14^, Limeira^14^, Luís Antônio^sc^, Mogi Guaçu^14^, Mongaguá^sc^, Pardinho^14^, Pariquera-Açú^sc^, Peruíbe*^,sc,14^, Piedade ^sc,14^, Piquete^14^, Piraju^14^, Praia Grande ^sc,14^, Ribeirão Branco^sc^, Ribeirão Grande^sc^, Rio Claro ^sc,14^, Rio Grande^14^, Salesópolis^sc,14^, Santo André^sc,14^, Santo Antônio do Pinhal^sc^, Santos^sc,14^, São Carlos^sc,14^, São José dos Campos^sc^, São Luís do Paraitinga^sc^, São Paulo ^sc,14^, São Roque^14^, São Sebastião*^,sc,14^, São Vicente ^sc,14^, Serra Negra^14^, Sete Barras^sc^, Sorocaba^sc^, Teodoro Sampaio^sc^, Ubatuba*^,sc,14^, Várzea Paulista^sc^, Vista Alegre do Alto^14^ |
| **Ceratophryidae** |  |
| *Ceratophrys aurita* | BA: Ilhéus^sc^, Irecê^sc^; ES: Conceição da Barra^sc^, Linhares^sc^; SP: Ribeirão Branco^sc^, Ubatuba^sc^ |
| **Craugastoridae** |  |
| *Haddadus binotatus* | BA: Camamu^sc^, Ibirapitanga^sc^, Ilhéus^sc^, Itabuna^sc^, Itacaré^sc^, Jequié^sc^, ES: Alegre^sc^, Aracruz^sc^, Cariacica^sc^, Conceição da Barra^sc^, Domingos Martins^sc^, Linhares^sc^, Mimoso do Sul^sc^, Muniz Freire^sc^, Santa Maria de Jetibá^sc^, Santa Teresa^sc^, São Mateus^sc^, Sooretama^sc^, Vargem Alta^sc^; MG: Congonhas^sc^, Lima Duarte^sc^, Matias Barbosa^sc^; PR: Antonina^sc^, Guaraqueçaba^sc^, Guaratuba^sc^, Morretes^sc^; RJ: Cachoeiras de Macacu^sc^, Engenheiro Paulo de Frontin^sc^, Nova Iguaçu^sc^, Parati^sc^, Rio de Janeiro^sc^, Santa Maria Madalena^sc^, Teresópolis^sc^; SC: Florianópolis^sc^, Governador Celso Ramos^sc^, São Bento do Sul^sc^, Treviso^sc^; SP: Angatuba^sc^, Arujá^sc^, Bertioga*^,sc^, Biritiba-Mirim^sc^, Cabreúva^sc^, Caieiras^sc^, Campinas^sc^, Cananéia^sc^, Caraguatatuba^sc^, Cubatão^sc^, Cunha^sc^, Eldorado^sc^, Guapiara^sc^, Guarujá^sc^, Iguape^sc^, Ilha Bela^sc^, Ilha Comprida^sc^, Iporanga^sc^, Itanhaém*^,sc^, Jacareí^sc^, Jacupiranga^sc^, Jundiaí^sc^, Lavrinhas^sc^, Mauá^sc^, Mogi das Cruzes^sc^, Pariquera-Açú^sc^, Peruíbe^sc^, Piedade^sc^, Pilar do Sul^sc^, Ribeirão Branco^sc^, Ribeirão Grande^sc^, Salesópolis^sc^, Santa Branca^sc^, Santana de Parnaíba^sc^, Santo André^sc^, Santos^sc^, São Bernardo do Campo^sc^, São Carlos^sc^, São Luís do Paraitinga^sc^, São Paulo^sc^, São Sebastião^sc^, São Vicente^sc^, Sete Barras^sc^, Ubatuba*^,sc^ |
| **Hemiphractidae** |  |
| *Fritziana fissilis* | MG: Passa Quatro^sc^; PR: Antonina^sc^, Guaraqueçaba^sc^, Morretes^sc^; RJ: Cachoeiras de Macacu^sc^, Itatiaia^sc^, Mangaratiba^sc^, Parati^sc^, Petrópolis^sc^, Teresópolis^sc^; SC: Apiúna^sc^, Florianópolis^sc^, Novo Horizonte^sc^; SP: Bananal^sc^, Bertioga*^,sc^, Biritiba-Mirim^sc^, Cotia^sc^, Cubatão^sc^, Cunha^sc^, Ilha Bela^sc^, Iporanga^sc^, Itanhaém^sc^, Juquitiba^sc^, Peruíbe^sc^, Piedade^sc^, Salesópolis^sc^, Santo André^sc^, São José do Barreiro^sc^, São Miguel Arcanjo^sc^, São Paulo, São Sebastião*^,sc^, Tapiraí^sc^, Ubatuba^sc^ |
| *Fritziana ohausi* | MG: Alto Caparaó^sc^; RJ: Itatiaia^sc^, Teresópolis^sc^; SP: Apiaí^sc^, Pilar do Sul^sc^, Ribeirão Grande^sc^, Salesópolis^sc^, Santo André^sc^, Santos^sc^, São José do Barreiro^sc^, São Luís do Paraitinga^sc^, Tapiraí^sc^ |
| **Hylidae** |  |
| *Aplastodiscus arildae* | ES: Castelo^15^, Domingos Martins^sc,15^; MG: Araponga^15^, Belo Horizonte ^sc,15^, Caparaó^sc^, Carangola^sc^, Catas Altas ^sc,15^, Itabirito^sc^, Lima Duarte^15^, Muriaé^15^, Nova Lima^sc^, Ouro Branco^15^, Ouro Preto^15^, Pedra Dourada^15^; RJ: Itatiaia ^sc,15^, Magé^sc^, Mangaratiba^15^, Nova Friburgo ^sc,15^, Santa Maria Madalena^sc^, Teresópolis ^sc,15^; SP: Bananal^sc^, Bertioga^sc^, Caçapava^sc^, Jundiaí ^sc,15^, Peruíbe^15^, Salesópolis ^sc,15^, Santo André^sc^, São José do Barreiro^sc^, São Luís do Paraitinga^sc^, São Paulo^sc^ |
| *Aplastodiscus eugenioi* | RJ: Angra dos Reis^sc,16^, Cachoeiras de Macacu^17^, Duque de Caxias^16^, Engenheiro Paulo de Frontin^16^, Guapimirim^17^, Itaguaí^sc^, Magé^17^, Mangaratiba^sc,16^, Nova Iguaçu^16^, Parati^16^, Rio de Janeiro^16^; SP: Caraguatatuba^sc,16^, Ilha Bela^16^, Ubatuba*^, sc, 16^ |
| *Aparasphenodon bokermanni* | SC: Guaramirim^18^; SP: Pariquera-Açú ^sc^, Cananéia^sc^, Iguape*^, sc^, Peruíbe ^sc^, Itanhaém* |
| *Aparasphenodon brunoi* | BA: Porto Seguro^20^; ES: Linhares^sc^, Pedro Canário^sc^, Sooretama^sc^, Vila Velha^sc^; MG: Marliéria^20^; RJ: Maricá^sc^, Rio de Janeiro^sc,20^; SP: Caraguatatuba^19^, Ubatuba^sc^ |
| *Dendropsophus berthalutzae* | ES: Santa Teresa^sc^; PR: Antonina^sc^, Guaraqueçaba^21^, Matinhos ^sc^, Morretes ^sc^; RJ: Itaguaí^sc^, Magé^sc,21^; SP: Bertioga*^,sc^, Biritiba-Mirim ^sc^, Brotas ^sc^, Cananéia ^sc^, Caraguatatuba ^sc^, Cubatão ^sc^, Iguape ^sc^, Ilha Bela ^sc^, Iporanga ^sc^, Itanhaém*^,sc^, Mogi das Cruzes ^sc^, Pariquera-Açú ^sc^, Peruíbe ^sc^, Registro ^sc^, Santo André ^sc^, Santos ^sc,21^, São Sebastião*, São Paulo ^sc^, Ubatuba*^,sc^ |
| *Dendropsophus decipiens* | BA: Cachoeira^sc^, Caravelas^sc^, Mata de São João^sc^, Salvador^sc^; ES: Aracruz^sc^, Conceição da Barra^sc^, Santa Teresa^sc^, São Mateus^sc^, Sooretama^sc^; MG: Matias Barbosa^sc^, Viçosa^sc^; PE: Bom Conselho^sc^, Igarassu^sc^; RJ: Itaguaí^sc^; SP: Cananéia*^,sc^, Iguape*^,sc^, Itanhaém*, Peruíbe* |
| *Dendropsophus elegans* | AL: Campo Alegre^sc^; BA: Aurelino Leal^sc^, Cachoeira^sc^, Camamu^sc^, Caraíva^sc^, Caravelas^sc^, Conceição da Barra^sc^, Eunápolis^sc^, Gandu^sc^, Ilhéus^sc^, Itabuna^sc^, Itagibá^sc^, Itamaraju^sc^, Jequié^sc^, Mucuri^sc^, Uruçuca^sc^; ES: Águia Branca^sc^, Alegre^sc^, Aracruz^sc^, Cachoeiro de Itapemirim^sc^, Cariacica^sc^, Domingos Martins^sc^, Governador Lindenberg^sc^, Guarapari^sc^, Linhares^sc^, Marataízes^sc^, Mimoso do Sul^sc^, Muniz Freire^sc^, Santa Leopoldina^sc^, Santa Teresa^sc^, Sooretama^sc^; MG: Araçuaí^sc^, Carangola^sc^, Caratinga^sc^, Matias Barbosa^sc^, Nanuque^sc^, Viçosa^sc^; PR: Adrianópolis^sc^, Antonina^sc^, Guaratuba^sc^, Morretes^sc^, São José dos Pinhais^sc^; PE: Recife^sc^; RJ: Duque de Caxias^sc^, Itatiaia^sc^, Magé^sc^, Petrópolis^sc^, Santa Maria Madalena^sc^, Seropédica^sc^, Teresópolis^sc^;: Garuva^sc^; SP: Apiaí^sc^, Arapeí^sc^, Arujá^sc^, Bertioga*^,sc^, Iguape*^,sc^, Iporanga^sc^, Itanhaém*^,sc^, Itariri^sc^, Jacupiranga^sc^, Juquiá^sc^, Nazaré Paulista^sc^, Pariquera-Açú^sc^, Peruíbe*^,sc^, Pindamonhangaba^sc^, Registro^sc^, Ribeirão Grande^sc^, Santo André^sc^, Santos^sc^, São José do Barreiro^sc^, São Luís do Paraitinga^sc^, São Miguel Arcanjo^sc^, São Paulo^sc^, São Sebastião*^,sc^, Ubatuba*^,sc^; SE: Itabaiana ^sc^, Laranjeiras ^sc^ |
| *Dendropsophus giesleri* | BA: Igrapiúna^22^, Porto Seguro^sc^; ES: Linhares^sc^, Santa Teresa^sc^; MG: Ouro Preto^23^; RJ: Duque de Caxias^17^, Guapimirim^24^, Magé^sc^; SP: Pedro de Toledo^sc^, Peruíbe^sc^, Ubatuba*^,sc^ |
| *Dendropsophus microps* | MG: Camanducaia^sc^; PR: Campina Grande do Sul^sc^, Jaguariaíva^sc^, Piraquara^sc^, São José dos Pinhais^sc^, Tijucas do Sul^sc^; RJ: Teresópolis^sc^; RS: Itati^sc^; SC: Angelina^sc^, Blumenau^sc^, Florianópolis^sc^, Garuva^sc^, Itapema^sc^, Luro Müller^sc^, São Bento do Sul^sc^, São Bonifácio^sc^; SP: Apiaí^sc^, Atibaia^sc^, Avaré^25^, Bananal^sc^, Biritiba-Mirim^sc^, Botucatu^25^, Campos do Jordão^sc^, Capão Bonito^sc^, Cunha^sc^, Eldorado^sc^, Guapiara^sc^, Iguape*^,sc^, Iporanga^sc^, Itanhaém*, Jundiaí^sc^, Pariquera-Açú^sc^, Peruíbe*, Piedade^sc^, Pilar do Sul^sc^, Ribeirão Branco^sc^, Ribeirão Grande^sc^, Salesópolis^sc^, Santa Isabel^sc^, Santo André^sc^, Santo Antônio do Pinhal^sc^, São José do Barreiro^sc^, São Luís do Paraitinga^sc^, Tapiraí^sc^ |
| *Dendropsophus minutus* | BOLIVIA: Cobija^sc^; BRAZIL: AL: Campo Alegre^sc^, Passo de Camaragibe^sc^; AM: Manaus^sc^, São Gabriel da Cachoeira^sc^; BA: Andaraí^sc^, Araçuaí^sc^, Barreiras^sc^, Cachoeira^sc^, Caraíbas^sc^, Entre Rios^sc^, Palmeiras^sc^, CE: Tianguá^sc^, Ubajara^sc^, ES: Águia Branca^sc^, Conceição da Barra^sc^, Domingos Martins^sc^, Linhares^sc^, Murici^sc^, Santa Teresa^sc^, São Mateus^sc^, Vargem Alta^sc^; GO: Alto Paraíso de Goiás^sc^, Baliza^sc^, Campo Limpo de Goiás^sc^, Formosa^sc^, Goiás^sc^, Itapirapuã^sc^, Mossâmedes^sc^, São João D'Aliança^sc^; MA: São Luís^sc^; MT: Alta Floresta^sc^, Barra do Garças^sc^, Brasnorte^sc^, Chapada dos Guimarães^sc^, Cuiabá^sc^, Itanhangá^sc^, Juína^sc^, Porto dos Gaéchos^sc^, Santa Terezinha^sc^, São José do Rio Claro^sc^, Tapurah^sc^, Vila Bela da Santíssima Trindade^sc^; MS: Aquidauana^sc^, Bodoquena^sc^, Bonito^sc^, Campo Grande^sc^, Costa Rica^sc^, Jardim^sc^, Três Lagoas^sc^; MG: Alpinópolis^sc^, Andradas^sc^, Belo Horizonte^sc^, Camanducaia^sc^, Caratinga^sc^, Congonhas^sc^, Fama^sc^, Jaboticatubas^sc^, Lagoa Santa^sc^, Lima Duarte^sc^, Nova Lima^sc^, Poços de Caldas^sc^, Santa Rita de Ibitipoca^sc^, Santana do Riacho^sc^, São Roque de Minas^sc^, Teófilo Otoni^sc^, Uberlândia^sc^, Vargem Bonita^sc^, Viçosa^sc^; PA: Canaã dos Carajás^sc^, Carajás^sc^, Curionópolis^sc^, Marabá^sc^, Oriximiná^sc^, Jacareacanga^sc^, Paranaíta^sc^, PR: Adrianópolis^sc^, Borba^sc^, Cianorte^sc^, Morretes^sc^, Ortigueira^sc^, Piraquara^sc^, São José dos Pinhais^sc^; PE: São Lourenço da Mata^sc^; PI: Piripiri^sc^; RJ: Cachoeiras de Macacu^sc^, Itaguaí^sc^, Magé^sc^, Nova Friburgo^sc^, Petrópolis^sc^, Resende^sc^, Santa Maria Madalena^sc^; RS: Bento Gonçalves^sc^, Cambará do Sul^sc^, Mato Castelhano^sc^, São Francisco de Paula^sc^, Sapiranga^sc^, Taquara^sc^, Viamão^sc^; SC: Bom Jardim da Serra^sc^, Chapecó^sc^, Lages^sc^, São Bento do Sul^sc^, São Domingos^sc^, Treviso^sc^; SP: Americana^sc^, Angatuba^sc^, Apiaí^sc^, Arapeí^sc^, Assis^sc^, Atibaia^sc^, Biritiba-Mirim^sc^, Botucatu^sc^, Brotas^sc^, Cabreúva^sc^, Cajamar^sc^, Campinas^sc^, Corumbataí^sc^, Cunha^sc^, Eldorado^sc^, Guapiara^sc^, Guareí^sc^, Ibiúna^sc^, Iguape*^,sc^, Iporanga^sc^, Itanhaém*^,sc^, Itapecerica da Serra^sc^, Itirapina^sc^, Jundiaí^sc^, Marília^sc^, Mauá^sc^, Miracatu^sc^, Mogi das Cruzes^sc^, Natividade da Serra^sc^, Nova Aliança^sc^, Ourinhos^sc^, Pedregulho^sc^, Peruíbe^sc^, Piracicaba^sc^, Pirassununga^sc^, Praia Grande^sc^, Ribeirão Branco^sc^, Ribeirão Grande^sc^, Ribeirão Preto^sc^, Rio Claro^sc^, Salesópolis^sc^, Santo André^sc^, Santo Antônio do Pinhal^sc^, São Carlos^sc^, São José do Rio Pardo^sc^, São Luís do Paraitinga^sc^, São José do Barreiro^sc^, São Paulo^sc^, São Sebastião*^,sc^, Sete Barras^sc^, Sumaré^sc^, Taubaté^sc^, Teodoro Sampaio^sc^, Ubatuba*^,sc^, Laranjeiras^sc^, TO: Araguaína^sc^, Caseara^sc^, Porto Nacional^sc^ |
| *Dendropsophus werneri* | PR: Adrianópolis^sc^, Antonina^sc^, Garuva^sc^, Guaratuba^sc^, Matinhos^sc^, Morretes^sc^, São José dos Pinhais^sc^; SC: Angelina^sc^, Barra Velha^sc^, Blumenau^sc^, Botuverá^sc^, Corupá^sc^, Navegantes^sc^, São Bento do Sul^sc^, São José^sc^; SP: Águas de Santa Bárbara^sc^, Apiaí^sc^, Barra do Turvo^sc^, Cananéia^sc^, Cubatão^sc^, Eldorado^sc^, Iguape*^, sc^, Ilha Comprida^sc^, Iporanga^sc^, Itanhaém*^, sc^, Mongaguá^sc^, Pariquera-Açú^sc^, Peruíbe*^, sc^, Praia Grande^c^, São Paulo^sc^, São Vicente^sc^ |
| *Hypsiboas albomarginatus* | AL: Campo Alegre^sc^, Ibateguara^26^, Maceió^26^, Murici^26^, Passo de Camaragibe^sc,26^, Quebrangulo^26^, Rio Largo^26^, São Miguel dos Campos^sc,26^; BA: Acajutiba^sc^, Aurelino Leal^sc^, Bonito^sc^, Cachoeira^sc^, Caraíbas^sc^, Caraíva^26^, Conde^26^, Entre Rios^26^, Feira de Santana ^sc,26^, Gandu ^sc^, Ilhéus ^sc,26^, Itabuna^26^, Itacaré ^sc^, Itagibá^26^, Jequié ^sc^, Jussari^26^, Maracás^26^, Mata de São João ^sc^, Porto Seguro^26^, Salvador ^sc,26^, Santa Cruz Cabrália^26^, Uruçuca ^sc,26^, Valença^26^; ES: Alegre ^sc^, Anchieta ^sc,26^, Aracruz ^sc^, Cariacica ^sc,26^, Domingos Martins ^sc,26^,Fundão ^sc^, Governador Lindenberg ^sc^, Guarapari ^sc^, Linhares ^sc,26^, Marataízes ^sc^, Mimoso do Sul ^sc,26^, Muniz Freire ^sc^, Santa Leopoldina^26^, Santa Teresa ^sc,26^; MG: Carangola ^sc^, Chiador^26^, São João Nepomuceno^26^, Teófilo Otoni ^sc,26^; PR: Guaraqueçaba^26^, Guaratuba ^sc^, Matinhos ^sc^; PE: Bonito ^sc^, Cabo de Santo Agostinho^26^, Caruaru^26^, Igarassu ^sc,26^, Jaqueira^26^, Recife ^sc,26^, Timbaúba^26^; RJ:Angra dos Reis ^sc,26^, Araruama^26^, Cabo Frio^26^, Campos dos Goytacazes^26^, Duque de Caxias ^sc,26^, Engenheiro Paulo de Frontin^26^, Itaguaí^26^, Magé^26^, Mangaratiba^26^, Maricá^26^, Niterói^26^, Nova Iguaçu^26^, Parati ^sc,26^, Resende^26^, Rio das Ostras^26^, Rio de Janeiro ^sc,26^, Santa Maria Madalena ^sc,26^, São João da Barra^26^, Saquarema^26^, Tanguá^26^, Teresópolis ^sc,26^; RN: Tibau do Sul ^sc^; SC: Blumenau ^sc^, Corupá ^sc,26^, Garuva ^sc^, Guaramirim^26^, Içara ^sc^, Itajaí^26^, Itapema ^sc^, Joinville^26^, Penha ^sc^, Porto Belo^26^; SP: Apiaí ^sc^, Arapeí ^sc^, Bertioga *^,sc,26^, Cananéia ^sc^, Caraguatatuba ^sc,26^, Cubatão ^sc,26^, Eldorado ^sc,26^, Ferraz de Vasconcelos^26^, Guarujá ^sc^, Iguape *^,sc,26^, Ilha Bela ^sc^, Ilha Comprida ^sc^, Ilha do Cardoso ^sc^, Iporanga ^sc,26^, Itanhaém*, ^26^, Itapetininga^26^, Itariri ^sc,26^, Jacupiranga ^sc,26^, Juquiá ^sc,26^, Miracatu^26^, Mogi das Cruzes ^sc^, Mongaguá ^sc^, Pariquera-Açú ^sc,26^, Peruíbe *^,sc^, Piedade ^sc^, Praia Grande^26^, Ribeirão Branco ^sc^, Santo André ^sc,26^, Santos ^sc,26^, São Luís do Paraitinga ^sc^, São Paulo ^sc,26^, São Sebastião *^,sc^, São Vicente ^sc^, Sete Barras ^sc,26^, Ubatuba *^,sc,26^; SE: Aracaju^26^, Areia Branca ^sc,26^, Cristinápolis^26^, Santa Luzia do Itanhy^26^, Santo Amaro das Brotas^26^, São Cristóvão^26^ |
| *Hypsiboas faber* | AL: Campo Alegre^sc^; BA: Aurelino Leal ^sc^, Bonito ^sc^, Camamu^sc^, Feira de Santana ^sc^, Itagibá ^sc^, Jequié ^sc^, Uruçuca ^sc^; ES: Conceição da Barra ^sc^, Domingos Martins ^sc^, Linhares ^sc^, Mimoso do Sul ^sc^, Santa Teresa ^sc^, Vargem Alta ^sc^; MG: Carangola ^sc^, Caratinga ^sc^, Congonhas ^sc^, Cristina ^sc^, Fama ^sc^, Itamonte ^sc^, Teófilo Otoni ^sc^, Viçosa ^sc^; PR: Adrianópolis ^sc^, Jaguariaíva ^sc^, Maringá ^sc^, Morretes ^sc^, Ortigueira ^sc^, Quatro Barras ^sc^, Tibagi ^sc^, Tijucas do Sul ^sc^, Wenceslau Braz ^sc^; RJ: Nova Friburgo ^sc^, Petrópolis ^sc^, Rio de Janeiro ^sc^, Santa Maria Madalena ^sc^; RS: Barracão ^sc^, Derrubadas ^sc^, São Francisco de Paula ^sc^, Sapiranga ^sc^; SC: Angelina ^sc^, Barra Velha ^sc^, Botuverá ^sc^, Campos Novos ^sc^, Corupá ^sc^, Lauro Müller ^sc^, Mafra ^sc^, São Bento do Sul ^sc^, Treviso ^sc^; SP: Analândia ^sc^, Angatuba ^sc^, Assis ^sc^, Bauru ^sc^, Bertioga*, Botucatu ^sc^, Campinas ^sc^, Eldorado ^sc^, Guareí ^sc^, Iguape*, Iporanga ^sc^, Itanhaém*, Itatiba ^sc^, Itirapina ^sc^, Jundiaí ^sc^, Luís Antônio ^sc^, Mairiporã ^sc^, Miracatu ^sc^, Natividade da Serra ^sc^, Nazaré Paulista ^sc^, Pedregulho ^sc^, Peruíbe*, Pilar do Sul ^sc^, Pindamonhangaba ^sc^, Queluz ^sc^, Ribeirão Grande ^sc^, Rio ClaroSP ^sc^, Santa Branca ^sc^, Santo AndréSP ^sc^, Santo Antônio do Pinhal ^sc^, São Bernardo do Campo ^sc^, São Carlos ^sc^, São José do Barreiro ^sc^, São Luís do Paraitinga ^sc^, São Paulo ^sc^, São Sebastião*, Sete Barras ^sc^, Teodoro Sampaio ^sc^, Ubatuba *^sc^ |
| *Hypsiboas raniceps* | AC: Cruzeiro do Sul ^sc,28^; AL: Jequiá da Praia ^27^, São Miguel dos Campos ^27^; AM: Beruri ^27^, Careiro ^27^, Codajás ^27^, Humaitá ^27^, Iranduba ^27^, Manacapuru ^27^, Manaus ^sc,28^, Urucará ^27^; BA: Barreiras ^27^, Chorrochó ^27^, Juazeiro ^sc,28^, Maracás ^27^, Queimadas ^27^, Xique-xique ^27^; CE: Crato ^27^; MA: Arari ^27^, Barra do Corda ^27^, São Pedro da Água Branca ^27^, Vargem Grande ^sc,28^, Vitória do Mearim ^27^; MT: Aquidauana ^27^, Chapada dos Guimarães ^27^, Cuiabá ^27^, Paranaíta ^sc,28^, Porto Estrela ^27^; MS: Campo Grande ^27^, Corumbá ^27^; MG: Araguari ^28^, Buritizeiro ^27^, Manga ^27^, Pirapora ^27^, Uberlândia ^28^; PA: Belém ^27^, Jacareacanga ^sc,28^, Juruti ^27^, Marajó ^27^, Oriximiná ^27^, Santarém ^27^, Taperinha ^27^, Tucuruí ^27^; PB: Areia ^27^, Caiçara ^27^, Campina Grande ^sc,28^, Coremas ^27^, Gurinhém ^27^, Mamanguape ^27^, São José do Bonfim ^sc,28^; PE: Bodocó ^27^, Exu ^27^, Recife ^27^; PI: Piripiri ^27^, Valença do Piauí ^27^; RN: Angicos ^27^, Natal ^27^; SP: Atibaia ^29^, Ilha Solteira ^sc,28^, Nova Aliança ^29^, Ourinhos ^sc,28^, Pariquera-Açú ^29^; SE: Brejo Grande ^27^, Santo Amaro das Brotas ^27^; TO: Formoso do Araguaia ^sc,28^ |
| *Hypsiboas semilineatus* | AL: Campo Alegre^sc^, Murici^30^, Rio Largo^30^; BA: Aurelino Leal^sc^, Camacan^sc^, Camamu^sc^, Caravelas^30^, Ilhéus^sc,30^, Itabuna^30^, Mata de São João^sc^, Mucuri^sc^, Porto Seguro^30^, Una^30^, Uruçuca^sc,30^, Valença^30^, Wenceslau Guimarães^30^; ES: Afonso Cláudio^30^, Aracruz^sc,30^, Cachoeiro de Itapemirim^sc,30^, Cariacica^sc^, Conceição da Barra^sc,30^, Domingos Martins^sc^, Guarapari^30^, Jaguaré^sc^, Linhares^sc^, Marataízes^30^, Mimoso do Sul^sc^, Rio Doce^30^, Santa Teresa^sc,30^, São Mateus^sc,30^, Sooretama^sc^; MG: Além Paraíba^30^, Belmiro Braga^30^, Caratinga^30^, Juiz de Fora^30^, Mar de Espanha^30^, Marliéria^30^, Matias Barbosa^sc^, Pirapetinga^30^, São João Nepomuceno^30^, Teófilo Otoni^sc^; PR: Antonina^30^, Guaratuba^sc^, Morretes^sc,30^; PE: Igarassu^sc^, Recife^30^, Timbaúba^30^; RJ: Angra dos Reis^30^, Araruama^30^, Barra do Piraí^30^, Barra Mansa^30^, Campos dos Goytacazes^30^, Duque de Caxias^sc,30^, Engenheiro Paulo de Frontin^30^, Itaperuna^30^, Magé^30^, Maricá^30^, Miguel Pereira^30^, Niterói^30^, Nova Iguaçu^30^, Petrópolis^30^, Piraí^sc,30^, Resende^30^, Rio Claro^30^, Rio de Janeiro^30^, Saquarema^30^, Brusque^30^; SC: Corupá^sc,30^, Guaramirim^30^, Itapema^sc^, Timbó^sc^; SP: Bananal^30^, Bertioga *^,sc,30^, Campinas^30^, Cananéia^sc^, Caraguatatuba^sc^, Cubatão^30^, Eldorado^sc,30^, Iguape *^,sc,30^, Ilha Comprida^sc^, Iporanga^sc^, Itanhaém *, Itariri^sc^, Juquiá^sc^, Mongaguá^sc^, Pariquera-Açú^sc,30^, Peruíbe *^,sc,30^, Piedade^sc^, Pindamonhangaba^sc^, Praia Grande^sc,30^, Santos^sc^, São Paulo^30^, São Sebastião *, Ubatuba *^,sc,30^ |
| *Itapotihyla langsdorffii* | BA: Ilhéus^sc^, Mata de São João^sc^, Uruçuca^sc^; ES: Conceição da Barra^sc^, Linhares^sc^, Santa Teresa^sc^, Sooretama^sc^; MG: Conceição do Mato Dentro^32^, Goianá^32^, Marliéria^32^, Rio Novo^32^, PR: Guaratuba^sc^, Matinhos^sc^; RJ: Parati^sc^, Rio de Janeiro^sc^; RS: Itati^33^; SP: Americana^sc^, Angatuba^sc^, Araçoiaba da Serra^sc^, Bertioga*^,sc^, Botucatu^sc^, Campos do Jordão^sc^, Cananéia^sc^, Cubatão^sc^, Guareí^sc^, Iguape*^,sc^, Ilha Comprida^sc^, Itanhaém*, Itirapina^sc^, Mogi Guaçu^sc^, Peruíbe*^,sc^, Registro^sc^, Ribeirão Grande^sc^, Rio Claro^sc^, Santos^sc^, São Joaquim da Barra^sc^, São José do Rio Pardo^sc^, São Sebastião*, Sumaré^sc^, Teodoro Sampaio^sc^, Ubatuba*^,sc^; SE: Santa Luzia do Itanhy^31^ |
| *Phyllomedusa distincta* | PR: Antonina^sc^, Bituruna^34^, Guaraqueçaba^sc^, ^34^, Guaratuba^sc^, Ibaiti^sc^, Matinhos^sc^; RS: Terra de Areia^sc^; SC: Barra Velha^sc^, Blumenau^sc^, Brusque^34^, Corupá^34^, Florianópolis^34^, Joinville^34^, Novo HorizonteSC^34^, Penha^sc^, Porto Belo^34^, Santo Amaro da Imperatriz^sc^, São Bento do Sul^sc^, ^34^, Timbó^34^, Treviso^sc^, Tubarão^sc^; SP: Apiaí^sc^, Capão Bonito^sc^, Eldorado^sc^, ^34^, Guapiara^34^, Iguape^sc^, ^34^, Iporanga^sc^, ^34^, ItanhaémSP*, Jacupiranga^sc^, ^34^, Miracatu^sc^, ^34^, Pariquera-Açú^sc^, Pedro de Toledo^34^, Peruíbe*, Pilar do Sul^sc^, Registro^sc^; Ribeirão Branco^sc^, ^34^; Ribeirão Grande^sc^; Sete Barras^sc^ |
| *Phyllomedusa rohdei* | BA: Aurelino Leal^sc^, Gandu^sc^, Ilhéus^sc^, Uruçuca^sc^; ES: Linhares^sc,35^, Santa Teresa^35^, Vargem Alta^35^; MG: Caratinga^35^, Marliéria^35^, Santana do Paraíso^35^; RJ: Duque de Caxias^35^, Itaguaí^sc^, Niterói^35^, Nova Iguaçu^35^, Rio de Janeiro^35^, São Pedro da Aldeia^35^, Saquarema^35^, Seropédica^sc,35^, Tanguá^35^, Teresópolis^35^; SP: São Sebastião^35^, Ubatuba^sc,35^ |
| *Scinax angrensis* | RJ: Angra dos Reis^36^, Mangaratiba^36^, Parati^36^; SP: Ubatuba^sc^ |
| *Scinax argyreornatus* | BA: Canavieiras^37^, Ilhéus^sc^, Itabuna^sc^, Itacaré^sc^, Mucuri^sc^, Porto Seguro^36^, Una^sc^; ES: Aracruz^sc^, Cariacica^sc^, Guarapari^sc^, Linhares^36^, Santa Teresa^sc, 37^, São Mateus^sc^, Vitória^sc^; PR: Matinhos^sc^, Morretes^sc^; RJ: Angra dos Reis^37^, Itaguaí^37^, Magé^36^, Maricá^sc^, Nova Iguaçu^sc^, Rio de Janeiro^sc^; SP: Bertioga*, Cananéia^sc^, Iguape*^,sc^, Ilha Comprida^sc^, Itanhaém*, Peruíbe*^,sc^, Registro^sc^, São Sebastião*^,sc^, Ubatuba*^,sc^ |
| *Scinax catharinae* | SC: Corupá^36^, Florianópolis^36^, Guaratuba^36^, São Bento do Sul^36^; SP:Bertioga^36^, Itanhaém*, São José do Barreiro^36^, Ubatuba* |
| *Scinax eurydice* | BA: Andaraí^sc^, Cachoeira^sc^, Caravelas^sc^, Feira de Santana^sc,38^, Gandu^sc^, Ilhéus^sc^, Itagibá^sc^, Itamaraju^sc^, Maracás^sc,39^, Mata de São João^sc^, Morro do Chapéu^sc,39^, Mucuri^sc^, Porto Seguro^sc^, Uruçuca^sc^; ES: Águia Branca^sc^, Aracruz^sc^, Cachoeiro de Itapemirim^sc^, Conceição da Barra^sc^, Guarapari^sc^, Linhares^sc^, Santa Teresa^sc^, São Mateus^sc^; MG: Carangola^sc^, Grão Mogol^sc^, Viçosa^sc^; PE: Paulista^sc^, São Lourenço da Mata^sc^; RJ: Parati^sc^, Petrópolis^sc^; SP: Mogi das Cruzes^sc^, Ubatuba*^,sc^ |
| *Scinax hayii* | ES: Cariacica^sc^, Santa Teresa^sc^, Vargem Alta^sc^; MG: Camanducaia^sc^, Poços de Caldas^sc^; RJ: Angra dos Reis^sc^, Itatiaia^sc^, Nova Friburgo^sc^, Parati^sc^, Petrópolis^sc^, Teresópolis^sc^; SC: Barra Velha^sc^, São Bento do Sul^sc^, São Francisco do Sul^sc^; SP: Arujá^sc^, Atibaia^sc^, Campos do Jordão^sc^, Cunha^sc^, Iguape^sc^, Ilha Bela^sc^, Iporanga^sc^, Itanhaém^sc^, Jundiaí^sc^, Mauá^sc^, Natividade da Serra^sc^, Peruíbe^sc^, Piedade^sc^, Piquete^sc^, Ribeirão Pires^sc^, Santo André^sc^, Santo Antônio do Pinhal^sc^, Santos^sc^, São José do Barreiro^sc^, São Luís do Paraitinga^sc^, São Paulo^sc^, São Sebastião^sc^, São Vicente^sc^, Suzano^sc^, Ubatuba^sc^ |
| *Scinax imbegue* | PR: Antonina^40^, Morretes^40^, Paranaguá^40^, Pontal do Paraná^40^; SC: Blumenau^40^, Brusque^40^, Guaramirim^40^, Itajaí^40^, Piçarras^40^; SP: Bertioga^40^, Cananéia^40^, Eldorado^40^, Iguape^40^, Itanhaém*^,40^, Peruíbe^40^, Santo André^40^ |
| *Scinax littoralis* | PR: Guaraqueçaba^21^, Morretes^sc^, Morretes^21^; SP: Bertioga^sc^, Iguape*^,sc^, Peruíbe*^,sc^, São Vicente^sc^ |
| *Scinax perereca* | RS: São Francisco de Paula^64^; SP: Apiaí^67^, Iporanga^67^, Ribeirão Branco^66^, Ubatuba^65^ |
| *Scinax perpusillus* | ES: Santa Teresa^sc^, Morretes^sc^; RJ:Campos dos Goytacazes^sc^, Rio de Janeiro^42^, Santa Maria Madalena^sc^; SP: Apiaí^sc^, Bertioga*, Biritiba-Mirim^sc^, Cananéia^sc^, Cubatão^sc^, Cunha^sc^, Iguape^sc^, Iporanga^sc^, Itanhaém*^,sc^, Mogi das Cruzes^sc^, Peruíbe^sc,41^, Pilar do Sul^sc^, Salesópolis^sc^, Santo André^sc^, São Paulo^sc,41^, São Sebastião*^,sc^, Tapiraí^sc^, Ubatuba^sc^,*^,41^ |
| *Scinax tymbamirim* | PR: Antonina^40^, Guaraqueçaba^40^, Morretes^40^, Pontal do Paraná^40^, São José dos Pinhais^40^; RJ: Maricá^40^, Rio de Janeiro^40^; RS: Arroio do Sal^40^, Guaíba^40^, Itati^40^, Santo Antônio da Patrulha^40^, São Franci^40^o de Paula^40^, Sapiranga^40^, Terra de Areia^40^, Torres^40^, Viamão^40^; ^40^: Águas Mornas^40^, Barra Velha^40^, Brusque^40^, Criciúma^40^, Florianópolis^40^, Lauro Müller^40^, Piçarras^40^, Rancho Queimado^40^, São Bento do Sul^40^, Timbé do Sul^40^, Treviso^40^; SP: Bertioga^40^, Cananéia^40^, Cubatão^40^, Iguape^40^, Jacupiranga^40^, Registro^40^, São Bernardo do Campo^40^, Ubatuba^40^ |
| *Scinax trapicheiroi* | RJ: Angra dos Reis^sc^, Mangaratiba^sc^, Maricá^sc^, Piraí^sc^, Rio de Janeiro^sc^, Saquarema^sc^; SP: Bertioga*, Iguape*, Itanhaém*, Ubatuba* |
| *Trachycephalus mesophaeus* | BA: Ilhéus^sc^, Jequié^sc^, Uruçuca^sc^; ES: Governador Lindenberg^sc^, Linhares^sc^, Santa Teresa^sc^; PR: Guaratuba^sc^, Morretes^sc^, Santa Maria Madalena^sc^; RS: Lajeado^sc^; SC: Blumenau^sc^, Botuverá^sc^, Corupá^sc^; SP: Bertioga*^,sc^, Cananéia^sc^, Caraguatatuba^sc^, Cubatão^sc^, Cunha^sc^, Eldorado^sc^, Iguape*^,sc^, Ilha Bela^sc^, Itanhaém*, Peruíbe*, Piedade^sc^, Santos^sc^, São Sebastião*, São Vicente^sc^, Ubatuba*^,sc^ |
| **Leptodactylidae** |  |
| *Physalaemus atlanticus* | SP: Bertioga*, Caraguatatuba^sc^, São Sebastião*, Ubatuba*^,sc, 43^ |
| *Physalaemus bokermanni* | SP: Bertioga*, ^sc^, Cubatão^sc^, Itanhaém*, Peruíbe*, Santo André^sc,45^, Santos^46^, São Bernardo do Campo^44^, São Paulo^sc^, São Sebastião*^,sc,46^ |
| *Physalaemus moreirae* | SP: Bertioga^47^, Caraguatatuba^45^, Guarujá^sc^, Ilha Bela^sc^, Itanhaém^sc^, Salesópolis^sc,45^, Santo André^sc^, Santos^sc,43^, São Paulo^sc^ |
| *Physalaemus spiniger* | PR: Guaraqueçaba^sc,43^, Paranaguá^sc^; SP: Apiaí^sc^, Cananéia^sc,43^, Caraguatatuba^43^, Eldorado^sc,48^, Iguape*^,sc,43^, Ilha Comprida^sc^, Iporanga^sc^, Itariri^sc^, Jacupiranga^sc^, Pariquera-Açú^sc^, Peruíbe*^,sc^, Rio Grande da Serra^48^ |
|  |  |
| *Leptodactylus latrans* | BOLIVIA: Santa Cruz de la Sierra^50^; BRAZIL: AC: Cruzeiro do Sul^sc^; AL: Campo Alegre^sc^, Porto Calvo^sc^; BA: Caetité^sc^, Camamu^sc^, Caravelas^sc^, Gandu^sc^, Ilhéus^sc^, Iraquara^sc^, Itabuna^sc^, Itagibá^sc^, Jequié^sc^, Lençóis^sc^, Manoel Vitorino^sc^, Mata de São João^sc^, Palmeiras^sc^, Porto Seguro^sc^, Una^sc^, Uruçuca^sc^; ES: Alegre^sc^, Cachoeiro de Itapemirim^sc^, Cariacica^sc^, Conceição da Barra^sc^, Fundão^sc^, Linhares^sc^, Mimoso do Sul^sc^, São Mateus^sc^, Sooretama^sc^, Vargem Alta^sc^, Vitória^sc^; GO: Anápolis^sc^, Baliza^sc^, Palmeiras de Goiás^sc^, Porangatu^sc^, Quirinópolis^sc^,São Simão^sc^; MA: Darcinópolis^sc^; MS: Corumbá^sc^; MG: Arantina^sc^, Arcos^sc^, Camanducaia^sc^, Fama^sc^, Jaboticatubas^sc^, Munhoz^sc^, Nanuque^sc^, Pirapora^sc^, Santana do Riacho^sc^, União de Minas^sc^; PA: Altamira^sc^, Canaã^sc^, Oriximiná^sc^, Maturéia^sc^; PR: Guaratuba^sc^, Ibaiti^sc^, Maringá^sc^, Matinhos^sc^; PE: Belém de São Francisco^sc^, Igarassu^sc^, Ilha de Itamaracá^sc^, Itacuruba^sc^, Sanharó^sc^; PI: São Raimundo Nonato^sc^; RJ: Maricá^sc^; RS: Dom Pedro de Alcântara^sc^, São Sepé^sc^; RO: Boa Vista^sc^; SC: Blumenau^sc^, Bom Jardim da Serra^sc^, Corupá^sc^, Içara^sc^, Lauro Müller^sc^, Treviso^sc^; SP: Angatuba^sc^, Apiaí^sc^, Arapeí^sc^, Assis^sc^, Bauru^sc^, Bertioga*^,sc^, Botucatu^sc^, Brotas^sc^, Campinas^sc^, Cananéia^sc^, Caraguatatuba^sc^,Corumbataí^sc^, Cubatão^sc^, Eldorado^sc^, Iguape*^,sc^, Ilha Comprida^sc^, Iporanga^sc^, Itanhaém*, Itirapina^sc^, Luís Antônio^sc^, Mairiporã^sc^, Natividade da Serra^sc^, Pariquera-Açú^sc^, Pedregulho^sc^, Peruíbe*^,sc^, Ribeirão Branco^sc^, Ribeirão Grande^sc^, Rio Claro^sc^, São Luís do Paraitinga^sc^, São Paulo^sc^, São Sebastião*^,sc^, São Vicente^sc^, Suzano^sc^, Ubatuba*^,sc^; TO: Colinas do Tocantins^sc^, Figueirópolis^sc^, Guaraí^sc^, Palmeirante^sc^, Porto Nacional^sc^, Tupiratins^sc^, Wanderlândia^sc^; VENEZUELA: Delta Amacuro^49^, Isla Margarita^49^; San Filipe^49^ |
| *Adenomera marmoratus* | PR: Bituruna^51^; RJ:Angra dos Reis^51^, Cachoeira de Macacu^sc^, Duque de Caxias^sc^, Ilha Grande^sc^, Itaguaí^sc^, Mangaratiba^sc^, Maricá^sc^, Rio de Janeiro^51^, Saquarema^sc^; SP: Arujá^sc^, Bertioga*, Biritiba-Mirim^sc^, Caieras^sc^, Cajamar^sc^, Campos do Jordão^sc^, Capão Bonito^sc^, Caraguatatuba^sc,51^, Cubatão^51^, Guarujá^sc^, Iguape^sc^, Ilha Bela^sc^, Iporanga^sc^, Itanhaém*, Itapetininga^51^, Jacareí^sc^, Mogi das Cruzes^sc^, Peruíbe^sc^, Ribeirão Grande^sc^, Salesópolis^sc^, Santa Isabel^sc^, Santana de Parnaíba^sc^, Santo André^sc^, São Luís do Paraitinga^sc^, São Miguel Arcanjo^sc^, São Paulo^sc^, São Sebastião^sc^, São Vicente^sc,51^, Sete Barras^sc^, Ubatuba^sc^, Santa Branca^sc^ |
| **Microhylidae** |  |
| *Arcovomer passarellii* | ES: Anchieta^60^, Aracruz^sc,60^, Aracruz^sc,60^, Linhares^60^, Serra^60^, Vitória^sc^; RJ: Arraial do Cabo^60^, Duque de Caxias^60^, Itaguaí^sc,60^, Rio das Ostras^60^, Rio de Janeiro^60^, Seropédica^sc^; SP: Santos^60^; Ubatuba*^,sc^ |
| *Myersiella microps* | ES: Alfredo Chaves^53^, Linhares^53^, Marechal Floriano^sc^, Santa Leopoldina^53^; MG: Caratinga, Cristina^sc^, Marmelópolis^sc^; RJ: Angra dos Reis^53^, Magé^sc^, Nova Friburgo^53^, Nova Iguaçu^sc,53^, Petrópolis^sc,53^, Rio de Janeiro^53^, Teresópolis^53^; SP: Caraguatatuba^sc^, Cubatão^53^, Ilha Bela^sc^, Piedade^sc^, Pilar do Sul^sc^, Piquete^sc^, Ribeirão Pires^53^, Salesópolis^53^, São José do Barreiro^sc^, Sete Barras^sc^, Ubatuba*^,sc^ |
| *Elachistocleis cesarii* | GO: Pontalina^57^; MT: São Félix do Araguaia^sc^; MG: Conceição do Mato Dentro^sc^, Ituiutaba^54^, Jaboticatubas^sc^, Munhoz^sc^, Nova Lima^sc^, Perdizes^54^, Santana do Riacho^sc^, Uberlândia^54^, Viçosa^sc^; RN: Macaíba^55^; SP: Angatuba^57^, Brotas^sc^, Cabreúva^sc^, Campinas^sc^, Campos do Jordão^sc^, Guareí^sc^, Piquete^56^, Piracicaba^sc^, Rio Claro^57^, São Luís do Paraitinga^sc^, São Paulo^sc^, Taubaté^sc^, Ubatuba^sc,57^ |
| *Chiasmocleis leucosticta* | PR: Morretes^sc^, São José dos Pinhais^sc^; SC: Caçador^sc^, Florianópolis^59^, Lauro Müller^sc^, São Bento do Sul^59^; SP: Apiaí^sc^, Barra do Turvo^59^, Bertioga^59^, Biritiba-Mirim^sc^, Cananéia^sc^, Capão Bonito^59^, Corupá^58^, Cotia^59^, Diadema^58^, Eldorado^59^, Guarujá^sc^, Iguape^sc^, Ilha Bela^58^, Iporanga^sc^, Itanhaém*, Peruíbe^59^, Piedade^sc,59^, Ribeirão Branco^58^, São Miguel Arcanjo^59^, São Sebastião^59^ |
| *Chiasmocleis lacrimae* | ES: Mucuri^58^, Porto Seguro^59^, Una^59^, Guarapari^sc,59^; RJ: Angra dos Reis^59^, Arraial do Cabo^59^, Cachoeiras de Macacu^59^, Duque de Caxias^59^, Itaguaí^sc,59^, Niterói^59^, Seropédica^sc,59^; SP: Bertioga^59^, Ilha Bela^sc^, Itanhaém*,Ubatuba* |
| **Odontophrynidae** |  |
| *Macrogenioglottus alipioi* | AL: Campo Alegre^sc^; BA:Uruçuca^sc^, ES: Santa Teresa^sc^; SP: Apiaí^sc^, Cotia^sc^, Iporanga^sc^, Piedade^sc^, Pilar do Sul^sc^, Ribeirão Branco^sc^, Ribeirão Grande^sc^, Ubatuba * |
| *Proceratophrys appendiculata* | RJ: Angra dos Reis^61^, Cachoeiras de Macacu^61^, Miguel Pereira^61^, Nova Friburgo^sc^, Nova Iguaçu^61^, Parati^sc,61^, Petrópolis^61^, Rio de Janeiro^61^, Teresópolis^sc,61^; SP: Bananal^61^, Caraguatatuba^sc^, Cunha^sc^, Natividade da Serra^sc^, São José do Barreiro^sc,61^, São Luís do Paraitinga^sc^, São Sebastião^sc^, Ubatuba^sc^ |
| *Proceratophrys* cf. *melanopogon* | MG: Araponga^61^, Bocaina de Minas^63^, Carangola^63^, Lima Duarte^62^, Passa Quatro^61^, Pedra Dourada^62^; RJ: Itatiaia^63^, Nova Friburgo^63^, Resende^63^, Rio de Janeiro^63^, Santa Maria Madalena^sc,63^; SP: Arapeí^sc^, Bananal^sc,63^, Bertioga*^,63^, Campos do Jordão^sc,63^, Cubatão^63^, Iguape^sc^, Itanhaém^sc,63^, Mongaguá^sc^, Natividade da Serra^sc^, Peruíbe^sc^, Pilar do Sul^sc^, Salesópolis^sc^, Santo André^sc,63^, Santo Antônio do Pinhal^63^, Santos^63^, São José do Barreiro^sc,63^, São José dos Campos^sc^, São Luís do Paraitinga^sc,63^, São Paulo^sc,63^, São Sebastião*^,63^ |

* Records from field work of present study

^SC^ Records from scientific collections

^1^ State Acronyms: AC: Acre; AL: Alagoas; AM: Amazonas; BA: Bahia; CE: Ceará; ES: Espírito Santo; GO: Goiás; MA: Maranhão; MG: Minas Gerais; MS: Mato Grosso do Sul; MT: Mato Grosso; PA: Pará; PB: Paraíba; PE: Pernambuco; PI: Piauí; PR: Paraná; RJ: Rio de Janeiro; RN: Rio Grande do Norte; RO: Roraima; RS: Rio Grande do Sul; SC: Santa Catarina; SP: São Paulo; SE: Sergipe; TO: Tocantins.

^2^ Verdade V.K., Rodrigues M.T., Cassimiro J., Pavan D., Liou N., & Lange M.C. (2008) Advertisement call, vocal activity, and geographic distribution of *Brachycephalus hermogenesi* (Giaretta and Sawaya, 1998) (Anura, Brachycephalidae). *Journal of Herpetology*, **42**, 542–549.

^3^ Canedo C. & Pimenta B.V.S. (2010) New species of *Ischnocnema* (Anura, Brachycephalidae) from the Atlantic Rainforest of the State of Espírito Santo, Brazil. *South American Journal of Herpetology*, **5**, 199–206.

^4^ Canedo C., Targino M., Leite F.S.F., & Haddad C.F.B. (2012) A new species of *Ischnocnema* (Anura) from the São Francisco Basin Karst Region, Brazil. *Herpetologica*, **68**, 393–400.

^5^ Pombal J.P. & Cruz C.A.G. (1999) Redescrição de *Eleutherodactylus bolbodactylus* (A. Lutz, 1925) e a posição taxonômica de E. gehrti (Miranda-Ribeiro, 1926) (Anura, Leptodactylidae). *Boletim do Museu Nacional*, **404**, 1–10.

^6^ Heyer W.R. (1984) Variation, systematics, and zoogeography of *Eleutherodactylus guentheri* and closely related species (Amphibia: Anura: Leptodactylidae). *Smithsonian Contributions to Zoology*, 402, 1–42 .

^7^ Kwet A. & Solé M. (2005) Validation of *Hylodes henselii* Peters , 1870 , from Southern Brazil and description of acoustic variation in *Eleutherodactylus guentheri* (Anura: Leptodactylidae). *Journal of Herpetology*, **39**, 521–532.

^8^ Campos J.R.C., Ananias F., Haddad C.F.B., & Kasahara S. (2008) Karyotypic similarity among *Barycholos ternetzi* and five species of the genus *Eleutherodactylus* from southeastern Brazil (Anura, Brachycephalidae). *Micron*, **39**, 151–9.

^9^ Siqueira C.C., Vrcibradic D., Dorigo T.A., & Rocha C.F.D. (2011) Anurans from two high-elevation areas of Atlantic Forest in the state of Rio de Janeiro, Brazil. *Zoologia*, **28**, 457–464.

^10^ Cruz C.A.G. & Fusinatto L.A. (2008) A new species of *Dendrophryniscus*, Jímenez de la Espada, 1871 (Amphibia, Anura, Bufonidae) from the Atlantic Rain Forest of Rio Grande do Sul, Brazil. *South American Journal of Herpetology*, **3**, 22–26.

^11^ Fouquet A., Recoder R., Teixeira M., Cassimiro J., Amaro R.C., Camacho A., Damasceno R., Carnaval A.C., Moritz C., & Rodrigues M.T. (2012) Molecular phylogeny and morphometric analyses reveal deep divergence between Amazonia and Atlantic Forest species of *Dendrophryniscus*. *Molecular phylogenetics and evolution*, **62**, 826–38.

^12^ Caramaschi U. & Pombal J.P. (2006) A new species of *Rhinella* Fitzinger, 1826 from the Atlantic Rain Forest, eastern Brazil (Amphibia, Anura, Bufonidae). *Papéis Avulsos de Zoologia*, **46**, 251–259.

^13^ Pramuk J.B. (2006) Phylogeny of South American *Bufo* (Anura: Bufonidae) inferred from combined evidence. *Zoological Journal of the Linnean Society*, **146**, 407–452.

^14^ Baldissera F.A., Caramaschi U., & Haddad C.F.B. (2004) Review of the *Bufo crucifer* species group, with descriptions of the two new related species (Amphibia, Anura, Bufonidae). *Arquivos do Museu Nacional Rio de Janeiro*, **62**, 255–282.

^15^ Silva-soares T., Valadares A.P., Koski D.A., Ferreira R.B., & Cruz C.A.G. (2011) New records and distribution of *Aplastodiscus arildae* (Cruz & Peixoto, 1985) (Amphibia, Anura, Hylidae) in the southeast of Brazil. *Herpetology Notes*, **4**, 255–258

^16^ Carvalho-e-silva A.M.P.T. & Carvalho-e-silva S.P. (2005) New species of the *Hyla albofrenata* group, from the states of Rio de Janeiro and São Paulo, Brazil (Anura, Hylidae). *Journal of Herpetology*, **39**, 73–81.

^17^ Salles R. de O.L., Weber L.N., & Silva-soares T. (2009) Amphibia, Anura, Parque Natural Municipal da Taquara, municipality of Duque de Caxias, state of Rio de Janeiro, southeastern Brazil. *CheckList*, **5**, 840–854.

^18^ Woehl-Jr G., & Woehl E.N. (2003) *Aparasphenodon bokermanni* – Geographic Distribution. *Herpetological Review*, **34**, 379.

^19^ Mollo-Neto A. & Teixeira M. (2012) Checklist of the genus *Aparasphenodon* Miranda-Ribeiro , 1920 ( Anura: Hylidae ): Distribution map , and new record from São Paulo state, Brazil. *CheckList*, **8**, 1303–1307.

^20^ Pimenta B.V.S., Napoli M.F., & Haddad C.F.B. (2009) A new species of casque-headed tree frog, genus *Apasphenodon* Miranda-Ribeiro (Amphibia: Anura: Hylidae) from the Atlantic Rainforest of sounthern Bahia, Brazil. *Zootaxa*, **2123**, 46–54.

^21^ Conte C.E., Garey M.V., Lingnau R., Silva M.X., Armstrong C., & Hartmann M.T. (2009) Amphibia, Anura, *Limnomedusa macroglossa*, *Dendropsophus anceps*, *D. berthalutzae*, *D. seniculus*, *Scinax littoralis*: new state records, distribution extension and filling gaps. *CheckList*, **5**, 202–209.

^22^ Camurugi F., Lima T.M., Mercês E.A., & Juncá F.A. (2010) Anurans of the Reserva Ecológica da Michelin, Municipality of Igrapiúna, State of Bahia, Brazil. *Biota Neotropica*, **10**, 305–312.

^23^ Fonseca R.A.M., Gonçalves M.A.F., & Nascimento L.B. (2011) New state record and distribution map of *Dendropsophus giesleri* (Mertens, 1950) in Serra do Espinhaço mountain range, Brazil. *Herpetology Notes*, **4**, 143–144.

^24^ Silva-Soares T., Hepp F., Costa P.N., Luna-Dias C. (2010) Anfíbios anuros da RPPN Campo Escoteiro Geraldo Hugo Nunes, Município de Guapimirim, Rio de Janeiro, Sudeste do Brasil. *Biota Neopropica*, **10**, 225–233.

^25^ Maffei F., Ubaid F., Almeida S., Rolim D.C., Scarpellini-Jr D.G., Moya G.M., Spirandelli-Cruz E.F., & Jim J. (2009) Amphibia, Anura, Hylidae, *Dendropsophus microps* (Peters, 1872): Distribution extension in state of São Paulo, Brazil and first record in Cerrado domain. *CheckList*, **5**, 776–779.

^26^ Carnaval A.C., Hickerson M.J., Haddad C.F.B., Rodrigues M.T., & Moritz C. (2009) Stability predicts genetic diversity in the Brazilian Atlantic forest hotspot. *Science*, **323**, 785–9.

^27^ Caramaschi U. & Niermeyer H. (2003) New species of the *Hyla albopunctata* group from central Brazil (Amphibia, Anura, Hylidae). *Boletim do Museu Nacional*, **504**, 1–8.

^28^ Carvalho T.R.D.E., Giaretta A.A., & Facure K.G. (2010) A new species of *Hypsiboas* Wagler (Anura: Hylidae) closely related to *H. multifasciatus* Günther from southeastern Brazil. *Zootaxa*, **2521**, 37–52.

^29^ Zina J., Sá F.P., & Prado C.A.P. (2010) Amphibia, Anura, Hylidae, *Hypsiboas raniceps* Cope, 1862: Distribution extension. *ChekList*, **6**, 230–231.

^30^ Caramaschi U., Pimenta B.V.S., & Feio R.N. (2004) Nova espécie do grupo de *Hyla geographica* Spix, 1984 da Floresta Atlântica, Brasil (Amphibia, Anura, Hylidae). *Boletim do Museu Nacional*, **518**, 1–14.

^31^ Arzabe C. & Loebmann D. (2006) Amphibia, Hylidae, *Itapotihyla langsdorffii*: distribution extension. *Checklist*, **2**, 33–34.

^32^ Kleinsorge J.M.D., Fonseca R.A.M., Pirani R.M., & Nascimento L.B. (2009) Amphibia, Anura, Hylidae, *Itapotihyla langsdorffii* (Duméril and Bibron, 1841): Correction of older record and distribution extention. *ChekList*, **5**, 876–878.

^33^ Lingnau R., Zank C., Colombo P., & Vinciprova G. (2006) Amphibia, Hylidae, *Itapotihyla* *langsdorffii*: distribution extension. *ChekList*, **2**, 38–39.

^34^ Pombal J.P. & Haddad C.F.B. (1992) Espécies de *Phyllomedusa* do grupo *burmeisteri* do Brasil oriental, com descrição de uma espécie nova (Amphibia, Hylidae). *Revista Brasileira de Biologia*, **52**, 217–229.

^35^ Caramaschi U. (2006) Redefinição do grupo *de Phyllomedusa hypochondrialis*, com redescrição de *P. megacephala* (Miranda-Ribeiro, 1926), revalidação de *P. azurea* Cope, 1862 e descrição de uma nova espécie (Amphibia, Anura, Hylidae). *Arquivos Do Museu Nacional. Rio De Janeiro*, **64**, 159–179.

^36^ Lourenço A.C.C., Nascimento L.B., & Pires M.R.S. (2009) A new species of the *Scinax catharinae* species group (Anura: Hylidae) from Minas Gerais , southeastern Brazil. *South American Journal of Herpetology*, **65**, 468–479.

^37^ Pombal J.P., Carvalho Jr R.R., Canelas M.A.S., & Bastos R.P. (2010) A new *Scinax* of the *S. catharinae* species group from Central Brazil (Amphibia: Anura: Hylidae). *Zoologia*, **27**, 795–802.

^38^ Bokermann W.C.A. (1968) Three New *Hyla* from the Plateau of Maracás, Central Bahia, Brazil. *Journal of Herpetology*, **1**, 25–31.

^39^ Nunes I., Carvalho-Jr R.R., & Pereira E.G. (2010) A new species of *Scinax* Wagler (Anura: Hylidae) from Cerrado of Brazil. *Zootaxa*, **2514**, 24–34.

^40^ Nunes I., Kwet A., & Pombal J.P. (2012) Taxonomic revision of the *Scinax alter* species complex (Anura: Hylidae). *Copeia*, **2012**, 554–569.

^41^ Bell R.C., Brasileiro C.A., Haddad C.F.B., & Zamudio K.R. (2012) Evolutionary history of *Scinax* treefrogs on land-bridge islands in south-eastern Brazil. *Journal of Biogeography*, **39**, 1733–1742.

^42^ Pombal J.P. & Bastos R.P. (2003) Vocalizações de *Scinax perpusillus* (A. Lutz & B. Lutz) e *S. arduous* Peixoto (Anura, Hylidae), com comentários taxonômicos. *Revista Brasileira de Zoologia*, **20**, 607–610.

^43^ Haddad C.F.B. & Sazima I. (2004) A new species of *Physalaemus* (Amphibia; Leptodactylidae) from the Atlantic forest in southeastern Brazil. *Zootaxa*, **12**, 1–12.

^44^ Cardoso A.J. & Haddad C.F.B. (1985) Nova espécies de *Physalaemus* do grupo *signiferus* (Amphibia, Anura, Leptodactylidae). *Revista Brasileira de Biologia*, **45**, 33–37.

^45^ Cruz C.A.G., Nascimento L.B., & Feio R.N. (2007) A new species of the genus *Physalaemus* Fitzinger, 1826 (Anura, Leiuperidae) from southeastern Brazil. *Amphibia-Reptilia*, **28**, 457–465.

^46^ Giaretta A.A., Martins L.B., & Santos M.P. (2009) Further notes on the taxonomy of four species of *Physalaemus* (Anura, Leiuperidae) from the Atlantic Forest of Southeastern Brazil. *Zootaxa*, **2266**, 51-60.

^47^ Provete D.B., Garey M. V., Dias N.Y.N., & Rossa-Feres D.C. (2011) The Tadpole of *Physalaemus* *moreirae* (Anura: Leiuperidae). *Herpetologica*, **67**, 258–270.

^48^ Nascimento L.B., Caramaschi U., & Cruz C.A.G. (2005) Taxonomic review of the species group of the genus *Physalaemus* Fitzinger, 1826 with revalidation of the genera *Engystomops* Jímezes-de-la-Espada, 1872 and *Eupemphix* Steindachner, 1863 (Amphibia, Anura, Leptodactylidae). *Arquivos do Museu Nacional Rio de Janeiro*, **63**, 297–320.

^49^ Barrio-Amorós C.L. (1998) Systematics and biogeography of the amphibians (Amphibia) of Venezuela. *Acta Biologica Venezuelica*, **18**, 1–93.

^50^ De La Riva I. & Maldonado M. (1999) First record of *Leptodactylus ocellatus* (Linnaeus, 1758) (Amphibia, Anura, Leptodactylidae) in Bolivian and comments on related species. *Graellsia*, **55**, 193–197.

^51^ Heyer W.R. (1973) Systematics of the marmoratus group of the frog genus *Leptodactylus* (Amphibia, Leptodactylidae). *Contributions in Science, Natural History Museum,* **251**, 1–50.

^52^ Feio R.N., Cruz C.A.G., & Izecksohn E. (2003) *Proceratophrys melanopogon* - Geographic Distribution. *Herpetological Review*, **34**, 163.

^53^ Nelson C.E. & Lescure J. (1975) The taxonomy and distribution of *Myersiella* and *Synapturanus* (Anura: Microhylidae). *Herpetologica*, **31**, 389–397.

^54^ Giaretta A.A., Andrade F.S. De, Haga I.A., & Carvalho D.L. (2012) An acoustic evaluation of the geographical distribution of *Elachistocleis cesarii* Miranda-Ribeiro 1920 (Anura, Microhylidae). *Herpetology Notes*, **5**, 375–383.

^55^ Magalhães F.D.E.M., Santana D.J., Neto A.M., & Garda A.A. (2012) The tadpole of *Elachistocleis cesarii* Miranda-Ribeiro , 1920. *Zootaxa*, **3187**, 54–56.

^56^ Nunes-de-Almeida C.H. & Toledo L.F. (2012) A new species of *Elachistocleis* Parker (Anura, Microhylidae) from the State of Acre, Northern Brazil. *Zootaxa*, **3424**, 43–50.

^57^ Toledo L.F., Loebmann D., & Haddad C.F.B. (2010) Revalidation and redescription of *Elachistocleis cesarii* (Miranda-Ribeiro, 1920) (Anura: Hylidae). *Zootaxa*, **60**, 50–60.

^58^ Cruz C.A.G., Caramaschi U., & Napoli M.F. (2007) A new species of *Chiasmocleis* (Anura, Microhylidae) from the Atlantic Rain Forest of northeastern Bahia, Brazil. *South American Journal of Herpetology*, **2**, 47–52.

^59^ Forlani M.C. (2010) Morphology of the genus Chiasmocleis Méhely, 1904 (Anura, Microhylidae, Gastrophryninae), and its phylogenetic implications*.Unpublished Master Thesis,* Universidade de São Paulo (USP), pp. 138.

^60^ Malagoli L.R., Condez T.H., & Haddad C.F.B. (2012) *Arcovomer passarellii* Carvalho, 1954 (Amphibia: Anura: Microhylidae) : Distribution extension in São Paulo state, Brazil and geographic distribution map. *CheckList*, **8**, 505–506.

^61^ Feio R.N., Cruz C.A.G., & Izecksohn E. (2003) *Proceratophrys melanopogon* - Geographic Distribution. *Herpetological Review*, **34**, 163.

^62^ Mângia S., Santana D.J., & Feio R.N. (2010) Advertisement Call of The Cycloramphid Toad *Proceratophrys melanopogon* (Miranda-Ribeiro, 1926). *South American Journal of Herpetology*, **5**, 127–131.

^63^ Prado G.M. & Pombal J.P. (2008) Espécies de *Proceratophrys* Miranda-ribeiro, 1920 com apêndices palpebrais (Anura; Cycloramphidae ). *Arquivos de Zoologia*, **39**, 1–85.

^64^ Kwet, A. and Di-Bernardo, M. 1999. *Pró-Mata - Anfíbios. Amphibien. Amphibians*. EDIPUCRS, Porto Alegre, Brazil.

^65^ Magrini L. & Giaretta A.A. (2010) Calls of two Brazilian species of *Scinax* of the *S. ruber* clade (Anura: Hylidae). Herpetology Notes, 3, 121–126.

^66^ Pombal J.P., Haddad C.F.B., & Kasahara S. (1995) A new species of *Scinax* (Anura: Hylidae) from Southeastern Brazil, with cmments on the genus. *Journal of Herpetology*, **29**, 1–6.

^67^ Araujo C.O. et al. (2010) Amphibians and reptiles of the Parque Estadual Turístico do Alto Ribeira (PETAR), SP : an Atlantic Forest remnant of Southeastern Brazil Amphibians and reptiles of the Parque Estadual Turístico do Alto Ribeira (PETAR), SP : an Atlantic Forest remnant. *BiotaNeotropica*, **10**, 0–18.
