# Supplementary material for: Geomorphology Drives Amphibian Beta Diversity in Atlantic Forest Lowlands of Southeastern Brazil
Source: PLoS One. 2016 May 12;11(5):e0153977. doi: 10.1371/journal.pone.0153977 (PMC4865194; doi:10.1371/journal.pone.0153977)
Supplement: S2 Table — Ampibian anuran species recorded in restinga forests from coastal plains of São Paulo state and its occurrence in four geomorphological units defined by Suguio and Martin ([40]; see Methods and Fig 1): Cananéia/Iguape, Itanhaém/Santos, Bertioga/São Sebastião and Ubatuba. (DOC) [file pone.0153977.s002.doc]

**Supporting Information**

**Geomorphology drives amphibian beta diversity**

**in Atlantic Forest lowlands of southeastern Brazil**

Amom Mendes Luiz, Thiago Augusto Leão-Pires & Ricardo J. Sawaya

**S2** **Species occurrences in four geomorphological units of study region**

| **Family** | Geomorphological unit | | | |
| --- | --- | --- | --- | --- |
| Species | Cananéia | Itanhaém | Bertioga | Ubatuba |
|  | Iguape | Santos | São Sebastião |  |
| **Brachycephalidae** |  |  |  |  |
| *Brachycephalus hermogenesi* | X | X | X | X |
| *Ischnocnema bolbodactyla* |  |  |  | X |
| *Ischnocnema guentheri* | X | X | X | X |
| *Ischnocnema parva* | X | X | X | X |
| **Bufonidae** |  |  |  |  |
| *Dendrophryniscus brevipollicatus* | X | X | X | X |
| *Dendrophryniscus leucomystax* | X | X | X | X |
| *Rhinella hoogmoedi* | X | X | X | X |
| *Rhinella icterica* | X | X | X | X |
| *Rhinella ornata* | X | X | X | X |
| **Ceratophryidae** |  |  |  |  |
| *Ceratophrys aurita* |  |  |  | X |
| **Craugastoridae** |  |  |  |  |
| *Haddadus binotatus* | X | X | X | X |
|  |  |  |  |  |
| **Family** | Geomorphological unit | | | |
| Species | Cananéia | Itanhaém | Bertioga | Ubatuba |
|  | Iguape | Santos | São Sebastião |  |
| **Hemiphractidae** |  |  |  |  |
| *Fritziana fissilis* | X | X | X | X |
| *Fritziana ohausi* | X | X | X |  |
| **Hylidae** |  |  |  |  |
| *Aplastodiscus arildae* | X | X | X | X |
| *Aplastodiscus eugenioi* |  |  |  | X |
| *Aparasphenodon bokermanni* | X |  |  |  |
| *Aparasphenodon brunoi* |  |  | X | X |
| *Dendropsophus berthalutzae* | X | X | X | X |
| *Dendropsophus decipiens* | X | X | X | X |
| *Dendropsophus elegans* | X | X | X | X |
| *Dendropsophus giesleri* | X | X | X | X |
| *Dendropsophus microps* | X | X | X | X |
| *Dendropsophus minutus* | X | X | X | X |
| *Dendropsophus werneri* | X | X |  |  |
| *Hypsiboas albomarginatus* | X | X | X | X |
| *Hypsiboas faber* | X | X | X | X |
| *Hypsiboas raniceps* | X |  |  |  |
| *Hypsiboas semilineatus* | X | X | X | X |
| *Itapotihyla langsdorffii* | X | X | X | X |
| *Phyllomedusa distincta* | X |  |  |  |
| *Phyllomedusa rohdei* |  |  |  | X |
| *Scinax angrensis* |  |  |  | X |
| *Scinax argyreornatus* | X | X | X | X |
| *Scinax catharinae* | X | X | X | X |
| *Scinax eurydice* |  |  |  | X |
| *Scinax hayii* | X | X | X | X |
| *Scinax imbegue* | X | X | X |  |
|  |  | | | |
| **Family** | Geomorphological unit | | | |
| Species | Cananéia | Itanhaém | Bertioga | Ubatuba |
|  | Iguape | Santos | São Sebastião |  |
| *Scinax littoralis* | X | X | X |  |
| *Scinax perpusillus* | X | X | X | X |
| *Scinax perereca* | X | X | X | X |
| *Scinax tymbamirim* | X | X | X | X |
| *Scinax trapicheiroi* | X | X | X | X |
| *Trachycephalus mesophaeus* | X | X | X | X |
| **Leptodactylidae** |  |  |  |  |
| *Physalaemus atlanticus* |  |  | X | X |
| *Physalaemus bokermanni* | X | X | X |  |
| *Physalaemus moreirae* | X | X | X | X |
| *Physalaemus spiniger* | X | X | X | X |
| *Leptodactylus latrans* | X | X | X | X |
| *Adenomera marmoratus* | X | X | X | X |
| **Microhylidae** |  |  |  |  |
| *Myersiella microps* | X | X | X | X |
| *Elachistocleis cesarii* |  |  |  | X |
| *Chiasmocleis leucosticta* | X | X | X | X |
| *Chiasmocleis lacrimae* | X | X | X | X |
| *Arcovomer passarellii* |  | X | X | X |
| **Odontophrynidae** |  |  |  |  |
| *Macrogenioglottus alipioi* | X |  |  | X |
| *Proceratophrys appendiculata* |  |  |  | X |
| *Proceratophrys* cf. *melanopogon* | X | X | X | X |
