# Supplementary material for: Geomorphology Drives Amphibian Beta Diversity in Atlantic Forest Lowlands of Southeastern Brazil
Source: PLoS One. 2016 May 12;11(5):e0153977. doi: 10.1371/journal.pone.0153977 (PMC4865194; doi:10.1371/journal.pone.0153977)
Supplement: S1 Text — Commented R Codes and link to data sources of all analyses. (PDF) [file pone.0153977.s003.pdf]

Geomorphology drives amphibian beta diversity in Atlantic Forest lowlands of southeastern Brazil

Luiz, A.M.; Leão-Pires, T.A.; Sawaya, R.J.

January 26 2016

Analytical Roadmap

Here we will detail and the roadmap of statiscal analysis from *Geomorphology drives amphibian beta diversity in Atlantic Forest lowlands of southeastern Brazil* (Luiz, Leao-Pires & Sawaya, 2016) submitted to PlosOne. We share the original data, codes and sources through this link.

Loading packages and data

Some packages may need to be installed directly from repository. You can use this code to install:

```
# install.packages("PACK_NAME", repos="http://R-Forge.R-project.org")
```

Loaging packages:

```
library(vegan)
library (ade4)
library (spacemakeR)
library (betapart)
library (packfor)
library (AEM)
library (sp)
library(HH)
library (maptools)
library (ggplot2)
source ("varpart.R")
source ("evplot.R")
```

We used several datasets to conduct our analysis, namely:

- Species incidencies table: binary table with incindence (1 or 0) of species (columns) in a given site (rows)
- Env: txt file with environmental variables extracted from worldclim
- XY: txt file with site names and their lat/long coordinates
- XY.km: coordinate sites in KM
- ge0: txt file with geomorphological units of sites
- colgeo: colors for geomorphological units

Loading Data Sets:

```r
# First:
# You must define the working directory with the files downloaded
#from the link shared above.

#setwd ('PATH WITH FILES')

# Loading and pre-processing data

Y<-read.table("Y_last.csv", header=T, row.names=1, sep=";")[,-c(1,2)]
Y<- decostand(Y, "hel") # Hellinger Transformation

Env<-read.table("E.txt", h=T, row.names=1)
env<-Env[,c(3,10,14,16)]

#This file contain the random selected sites used in the paper.
#As specified in the paper, we neede randomly sample sites from
#Cananeia/Iguape geomorphological unit that has disproportionally more sites.
rand.Y<-as.matrix(read.table("rand.txt", h=T, sep=','))[,-1]

# Reading the coordinates data
xy<-read.table("XY.txt", h=T, row.names=1)
xy.km <-read.table('xykm.txt', h=T, row.names=1)
sites<-rownames(Y)

ge0<-read.table("geo.txt", h=T, row.names=1)
ge0_dummy<-read.table("dummy.csv", h=T, row.names=1, sep=";")
ge0[70:72,2]<-4
ge0<-model.matrix(~ge0$Geology, data=ge0)[,-1]
rownames(ge0)<- rownames(Env)
colnames (ge0)<-c("Santos", "Sao_Sebastiao", "Ubatuba")

colgeo<-as.data.frame(read.table("colgeo.csv", h=T, row.names=1, sep=";"))
colgeo[70:72,1]<-"Cananéia/ Iguape"
rownames(colgeo)<- rownames(Env)

# Reading shape of study area
map<- readShapeSpatial("BRA_adm1_Select.shp")

# Function to load some specifications to plot the study area
plotmap <- function(map)
{
  par(mar = rep(0.1, 4))
  plot(map, xlim=c(-48.2,-44.85),ylim=c(-24.8,-23.1), col ='grey80',
       pbg ='grey40', bg ='grey40', border ='grey40')
}
```

```r
# Subsetting data

Y<-Y[rand.Y,]

xy.km<-xy.km[rand.Y,]
xy<-xy[rand.Y,]
colnames (xy.km)<- c("Long", "Lat")
```

```r
dist.xy<- dist(xy.km)

ge0<-ge0[rand.Y,]
ge0_dummy<-ge0_dummy[rand.Y,]
rownames(ge0)<-rownames(Y)

colgeo<-colgeo[rand.Y,]
rownames(colgeo)<-rownames(Y)
colnames (colgeo)<- c("Unit", "Number")
colgeo<-colgeo[,2]
env<- env[rand.Y,]
Env<- Env[rand.Y,]

# Map from study region
map<- readShapeSpatial("BRA_adm1_Select")
plotmap <- function()  # setting map attributes
{
  par(mar = rep(0.1, 4))
  plot(map, xlim=c(-48.2,-44.85),ylim=c(-24.8,-23.1),
    col ='grey80', pbg ='grey40',
    bg ='grey40', border ='grey40')
}
```

Our strategy to select environmental variables were firstly remove the correlated ones at r<.85 and select those variables with biological meaning for anurans distribution. After this first approach, we use a PCA to extract orthogonal axes that represent variation in the climatic data, since that data still has some degree of correlation:

```r
# PCA
pca.env<- rda (env, scale = TRUE)
# Scale = TRUE define that PCA is based on correlations

# The proportion explained of axes:
pca.env$CA$eig * 100 / sum(pca.env$CA$eig)
```

```
##       PC1       PC2       PC3       PC4
## 67.448066 24.441093  4.323897  3.786945
```

```r
cumsum(pca.env$CA$eig * 100 / sum(pca.env$CA$eig))
```

```
##       PC1       PC2       PC3       PC4
##  67.44807  91.88916  96.21306 100.00000
```

We explore more two criteria that could be used to select PCA Axes, from Borcard et al. 2011 (pg. 121 - 123)

```r
# Eigenvalues from PCA greater than mean eigenvalue
ev<-mean(pca.env$CA$eig)
pca.env$CA$eig > mean (pca.env$CA$eig)
```

```
##   PC1   PC2   PC3   PC4
##  TRUE FALSE FALSE FALSE
```

```r
#Plotting PCA Eigenvalues related to Broken Strick model and Kaiser Guttman criterion
source ("evplot.R")
evplot (pca.env$CA$eig)
```

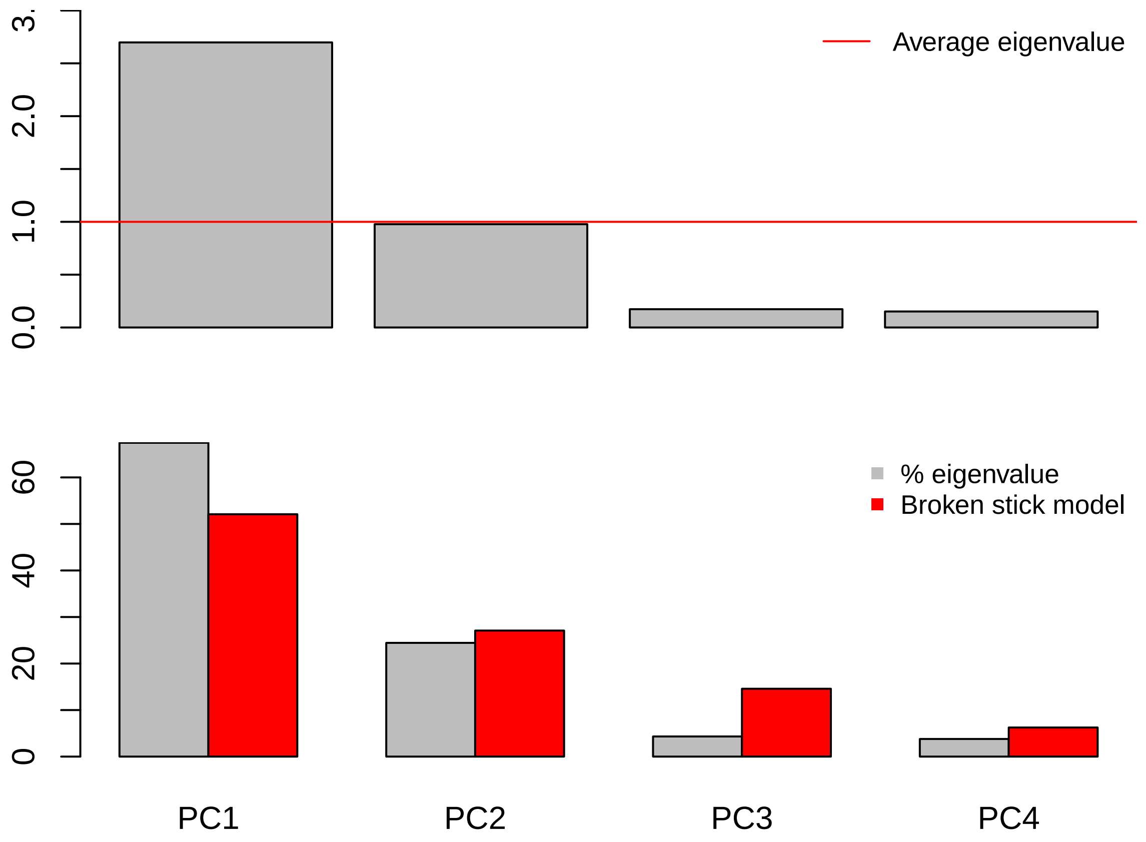

Then, the first axis explained ~ 67% of environmental data variance, the second one ~25%, the third and fourth, 4.3% and 3.8%, respectvely.

Based on proportion explained by each PCA axis, we have choosen to retain the first two axes, which explained ~92% of variance of climatic data. We have choosen to retain the first two axis, because by excluding the second one, we would be loosing substantial information about variation in the climatic data (~25%)

```r
#Next command provides loadings of environmental variables in relation to these  two axis:
sco<-pca.env$CA$v[,c(1,2)]
sco[order(sco[,1]),]
```

```
##                           PC1        PC2
## PrecDriestQua    -0.5290386 -0.3869014
## MeanColdestMonth -0.4196234  0.6959346
## PrecSeasonality   0.4915500  0.5212348
## Seasonality       0.5499209 -0.3070774
```

```r
pca.env<-scores (pca.env)$sites

# The first and last rows of PCA Environamental Axis
head (pca.env);tail (pca.env)
```

```
##               PC1       PC2
## Ub_26 -0.4068158 0.3652588
## Ub_27 -0.3944405 0.3729230
## Ub_28 -0.5438239 0.4027740
## Ub_30 -0.6740060 0.4039577
## Ub_13 -0.1983606 0.4409759
## Ub_16 -0.2423516 0.4768384
```

```
##                  PC1          PC2
## CnIg_266 0.06418733 -0.747912534
## CnIg_123 0.26683323 -0.230378544
## CnIg_137 0.54698503 -0.004842165
## CnIg_214 0.45110218 -0.198674182
## CnIg_296 0.24542162 -0.036777692
## CnIg_32  0.61225093  0.048112075
```

We also removed linear trends in the data (see more details in Methods of the paper)

```
anova(rda(Y~., data=as.data.frame(xy)))
```

```
## Permutation test for rda under reduced model
## Permutation: free
## Number of permutations: 999
##
## Model: rda(formula = Y ~ X_CENTROID + Y_CENTROID, data = as.data.frame(xy))
##           Df Variance      F Pr(>F)
## Model      2 0.063305 67.481  0.001 ***
## Residual 161 0.075518
## ---
## Signif. codes:  0 '***' 0.001 '**' 0.01 '*' 0.05 '.' 0.1 ' ' 1
```

```
Y.det<-resid(lm(as.matrix(Y)~., data=xy))
decorana(downweight(Y))
```

```
##
## Call:
## decorana(veg = downweight(Y))
##
## Detrended correspondence analysis with 26 segments.
## Rescaling of axes with 4 iterations.
## Downweighting of rare species from fraction 1/5.
##
##                     DCA1    DCA2     DCA3     DCA4
## Eigenvalues       0.1236 0.04515 0.017672 0.034939
## Decorana values   0.1406 0.02174 0.007602 0.005694
## Axis lengths      1.3215 0.93005 0.497471 0.743936
```

```
# Checking data

sum(rownames(Y) == rownames (env)); sum(rownames(Y) == rownames (ge0))
```

```
## [1] 164
```

```
## [1] 164
```

MEMs approach to Spatial Predictors

To create spatial predictors we implemented the method develeloped by Dray et al.(2006).The codes were adapted from Dray et al.(2006) and Borcard et al. (2011).

```
#Thresholds and nb object
max(dist(xy.km))
```

```
## [1] 386.7349
```

```
# Vector of thresholds distances
thresh<- seq(give.thresh(dist(xy.km)), 250, le=50)
list.nb<- lapply (thresh, dnearneigh, x=as.matrix (xy.km), d1=0)

# Neighbourhood matrices
list.nb[[1]]# Change this index and you will see any of the nb matrices created above
```

```
## Neighbour list object:
## Number of regions: 164
## Number of nonzero links: 2582
## Percentage nonzero weights: 9.599941
## Average number of links: 15.7439
```

```
# Let's see the first nb matrix
plotmap()
plot (list.nb[[1]], xy, add=T)
```

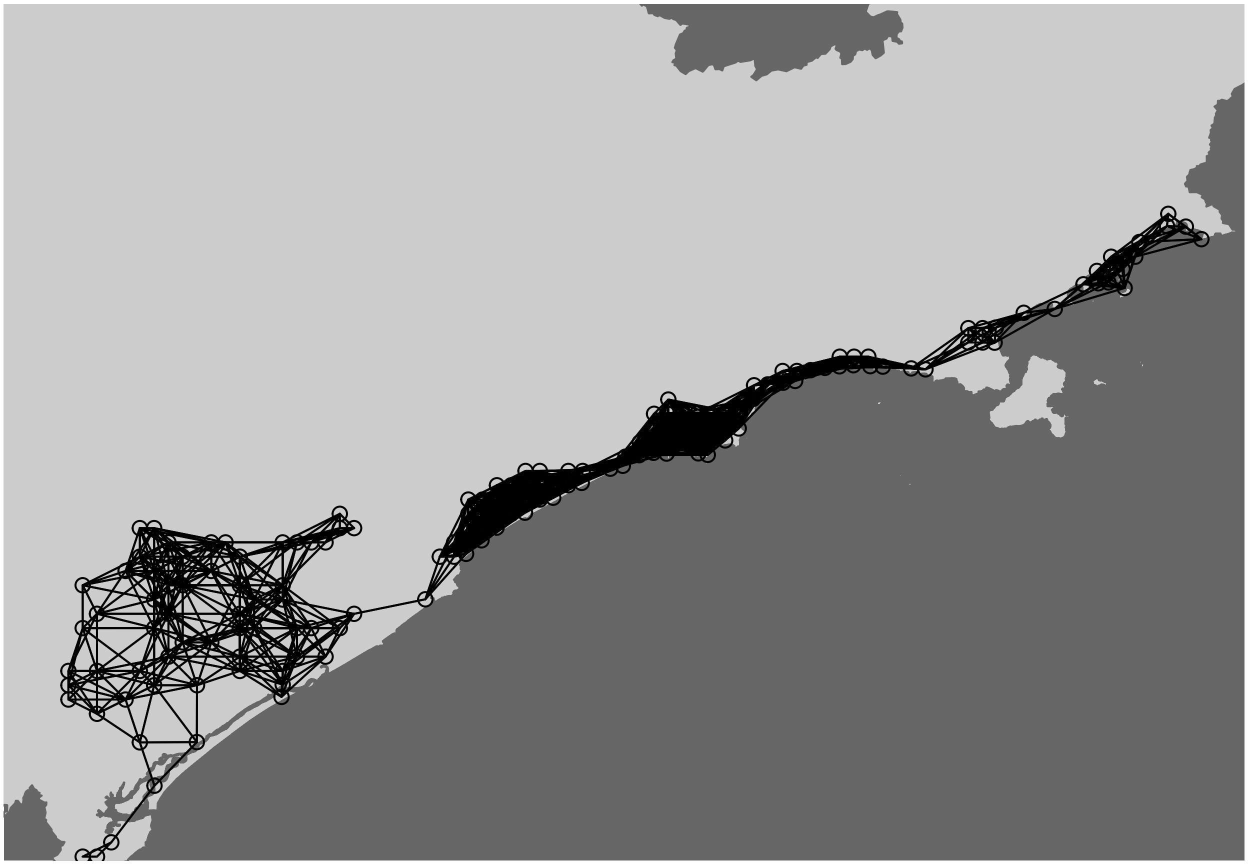

To assess the best spatial model, we used the *test.W* function from spacemakeR packgage:

```
#Y.W<- lapply (list.nb, test.W, Y=Y.det, xy=xy.km, MEM.autocor='positive')
```

We isolated the chunk code that run this analysis to simplify this document.

Now, we can extract the best spatial model, based on AICc:

```
minAIC<- sapply(Y.W, function (x) min(x$best$AICc, na.rm=T))
min(minAIC) # Smallest AICc
```

```
## [1] -643.5108
```

```
range(minAIC)
```

```
## [1] -643.5108 -432.7504
```

```
which.min(minAIC) # Number of the best model among 50 tested
```

```
## [1] 1
```

```
thresh[which.min(minAIC)] # Distance of best model among 20
```

```
## [1] 22.36665
```

```
R2.list<-Y.W[[2]]$best$R2[which.min(Y.W[[2]]$best$AICc)]

# MEM's selected by the best spatial model
# (smallest AICc) through test.W

MEMid<- Y.W[[which.min(minAIC)]]$best$ord[1:
    which.min(Y.W[[which.min(minAIC)]]$best$AICc)]
sort(MEMid)
```

```
##  [1]  1  2  3  4  5  6  7  8  9 10 11 12 13 14 15 17 18 19 20 22 23 25
```

```
MEM.all<-Y.W[[which.min(minAIC)]]$best$vectors
eigen.all<-Y.W[[which.min(minAIC)]]$best$values
eigen.all[eigen.all<mean(eigen.all)]
```

```
##  [1]  3.06679877  2.55308200  2.32322897  1.96481789  1.80605413
##  [6]  1.65234365  1.51018000  1.46083607  1.17287120  1.12843066
## [11]  1.08946157  0.80649783  0.76596444  0.75739101  0.63615636
## [16]  0.51395121  0.50045245  0.44008844  0.38139309  0.36413108
## [21]  0.34665624  0.25656418  0.24934475  0.22019059  0.13201546
## [26]  0.09575327  0.06231942  0.03895619 -0.04668157 -0.05184056
## [31] -0.06419044 -0.07840420
```

```
MEM.selected<-Y.W[[which.min(minAIC)]]$best$vectors[,sort(c(MEMid))]

eigen.sel<-eigen.all[sort(c(MEMid))]
colnames(MEM.selected)<-sort(MEMid)
MEMs<- MEM.selected
colnames(MEMs) <- paste("MEM", colnames(MEMs))
```

Lets see MEM values in the space. The values represent the scores of spatial eigenvectors created.

```
par(mfrow=c(1,2))
plotmap()
  s.value(xy, MEM.selected[,1],csize=0.5, pch=22,
    csub=2,xlim=c(-48.5,-44.5),ylim=c(-25.5,-23),
    clegend=.8, add.plot=T)

plotmap()
  s.value(xy, MEM.selected[,20],csize=0.5, pch=22,
    csub=2,xlim=c(-48.5,-44.5),ylim=c(-25.5,-23)
    ,clegend=.8, add.plot=T)
```

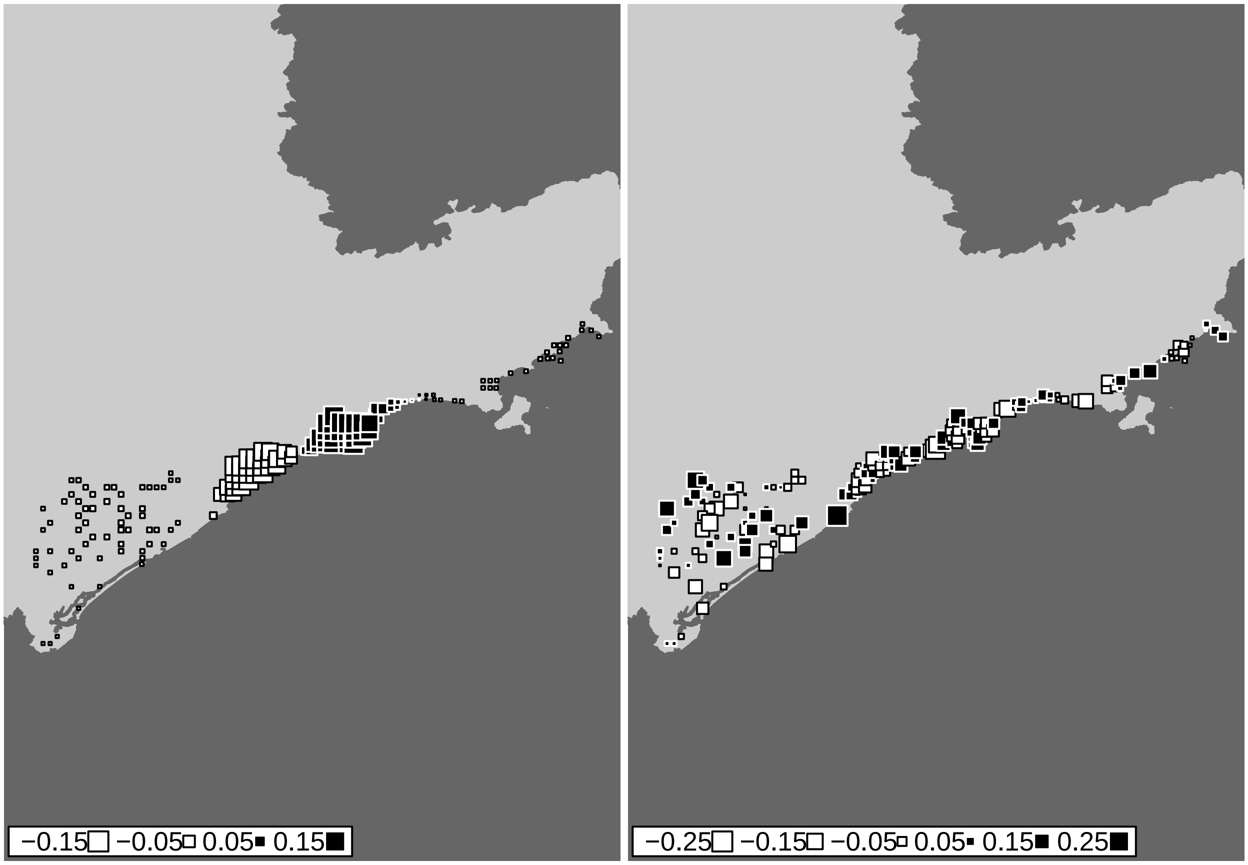

We refine the spatial model with a stepwise selection (forward) with double criteria:

```
# To refine the spatial model we run a stepwise procedure with double
# criteria from Blanchet et al.
```

```r
spatial<-MEM.all # positive eigenvalues
Y1<-rda (Y.det~., data=as.data.frame(spatial))
R2.spatial<- RsquareAdj(Y1)
forward<- forward.sel(Y.det, spatial,
    R2thresh=R2.spatial$r.squared,
        adjR2thresh=R2.spatial$adj.r.squared, nperm=999)
```

```
## Testing variable 1
## Testing variable 2
## Testing variable 3
## Testing variable 4
## Testing variable 5
## Testing variable 6
## Testing variable 7
## Testing variable 8
## Testing variable 9
## Testing variable 10
## Testing variable 11
## Testing variable 12
## Testing variable 13
## Testing variable 14
## Testing variable 15
## Testing variable 16
## Testing variable 17
## Testing variable 18
## Testing variable 19
## Testing variable 20
## Testing variable 21
## Procedure stopped (adjR2thresh criteria) adjR2cum = 0.778263 with 21 variables (superior to 0.778070)
```

```r
MEMs.fwd<-sort(forward$order)
MEMs.fwd<-spatial[,c(MEMs.fwd)]

# As described in the paper, we excluded the MEMs correlated with
#geomorphological variables

# MEMs scales:
# These are Broad Scale MEMs that are not correlated with Geomorphology

broad<-MEMs.fwd[,c(1,5,7,8,9,11)]
colnames(broad) <- paste("BS", 1:ncol(broad))

# These are the Fine Scale MEMs

fine<-MEMs[,12:ncol(MEMs.fwd)]
colnames(fine) <- paste("FS", 1:ncol(fine))
```

Variation Partitioning Approach

Now we have the climatic and spatial datasets to produce the model and variation partitioning analysis

In order to address some questions we are facing about model construction, we'll assess more than one model to variation partitioning to support the model selected…

```r
# First we will assess the Y variance explained by PCA Axes
#  (Enviromental), Geomorphology and Spatial variables in Broad and Fine scales

plot(varpart4.MEM(Y.det, pca.env, ge0, broad, fine,
        is.MEM=c(3,4), method='hierarchical'))
```

```
## Files X3 and X4 contain MEM eigenfunctions
## Hierarchical partitioning of the shared fractions
```

```r
title('Y ~ Env.PCA (X1) + Geo (X2) + Broad (X3) + Fine (X4)')
```

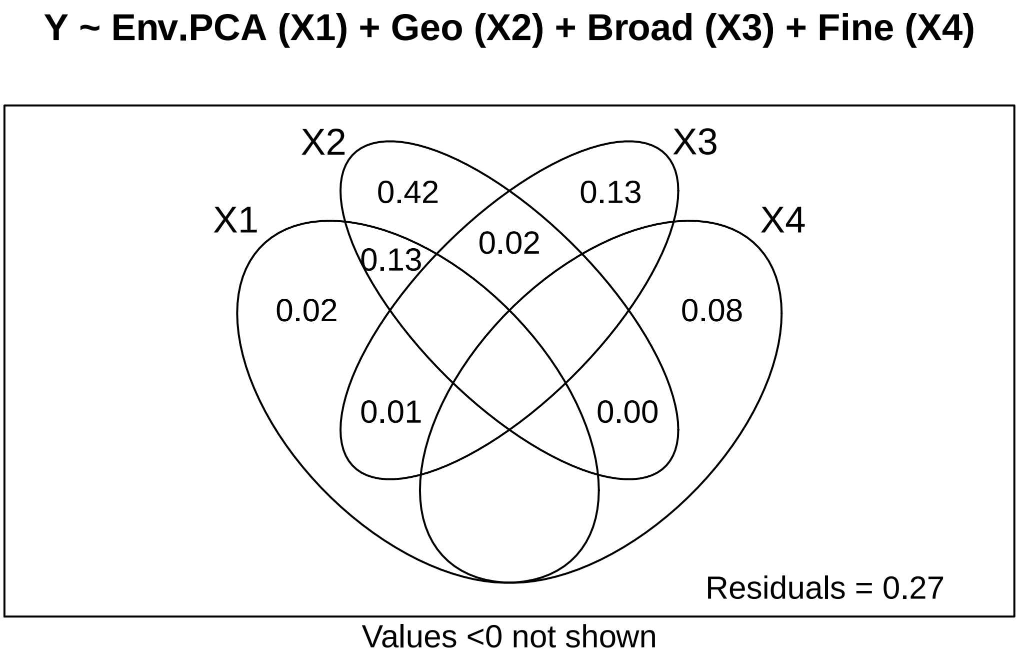

The graph above represent the variation partitioning with the Environmental PCA axis

```r
# Here we will assess the Y variance explained by Raw Enviromental Variables,
#Geomorphology and Spatial variables in Broad and Fine scales:

plot(varpart4.MEM(Y.det, env, ge0, broad, fine, is.MEM=c(3,4), method='hierarchical'))
```

```
## Files X3 and X4 contain MEM eigenfunctions
## Hierarchical partitioning of the shared fractions
```

```r
title(main = 'Y ~ Env Raw (X1) + Geo (X2) + Broad not Correlated (X3) + Fine (X4)')
```

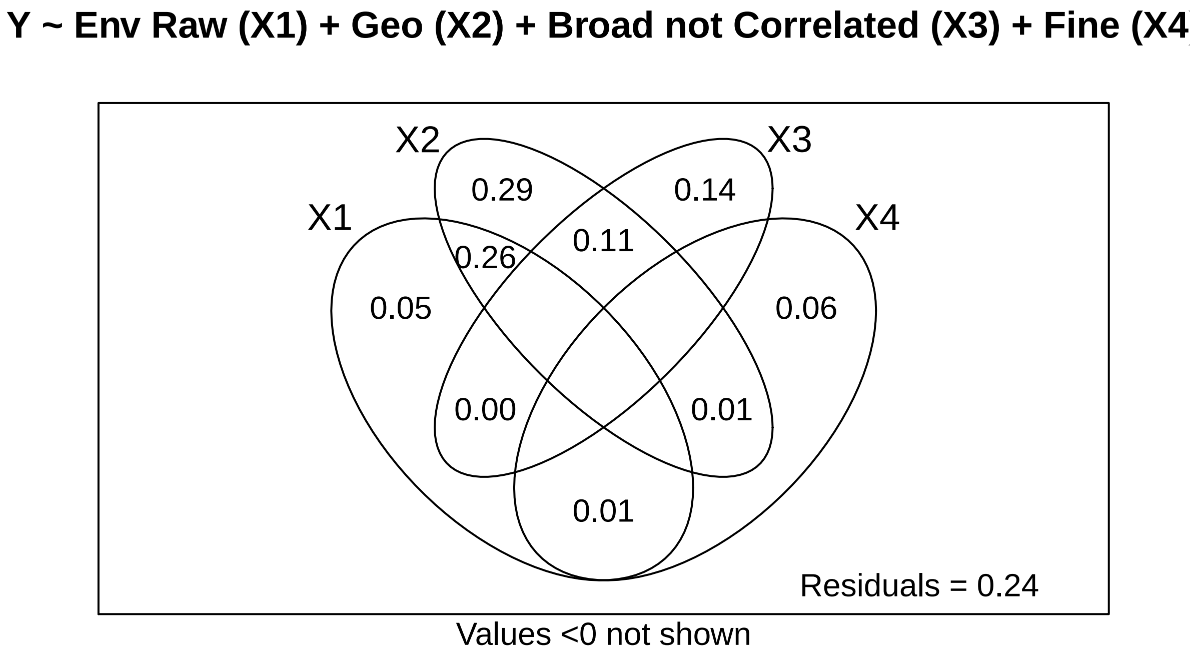

The graph above represent the variation partitioning with raw environmental variables

```
# Here we will assess the Y variance explained by PCA Axes (Enviromental),
#Geomorphology and Spatial variables in Broad and Fine scales.
# But, in this model we included those MEMs correlated with Geomorphology:

plot(varpart4.MEM(Y.det, pca.env, ge0, MEMs.fwd[,1:11],
 MEMs.fwd[,12:ncol(MEMs.fwd)], is.MEM=c(3,4),
    method='hierarchical'))
```

```
## Files X3 and X4 contain MEM eigenfunctions
## Hierarchical partitioning of the shared fractions
```

```
title(main = 'Y ~ Env.PCA (X1) + Geo (X2) + All Broad (X3) + Fine (X4)')
```

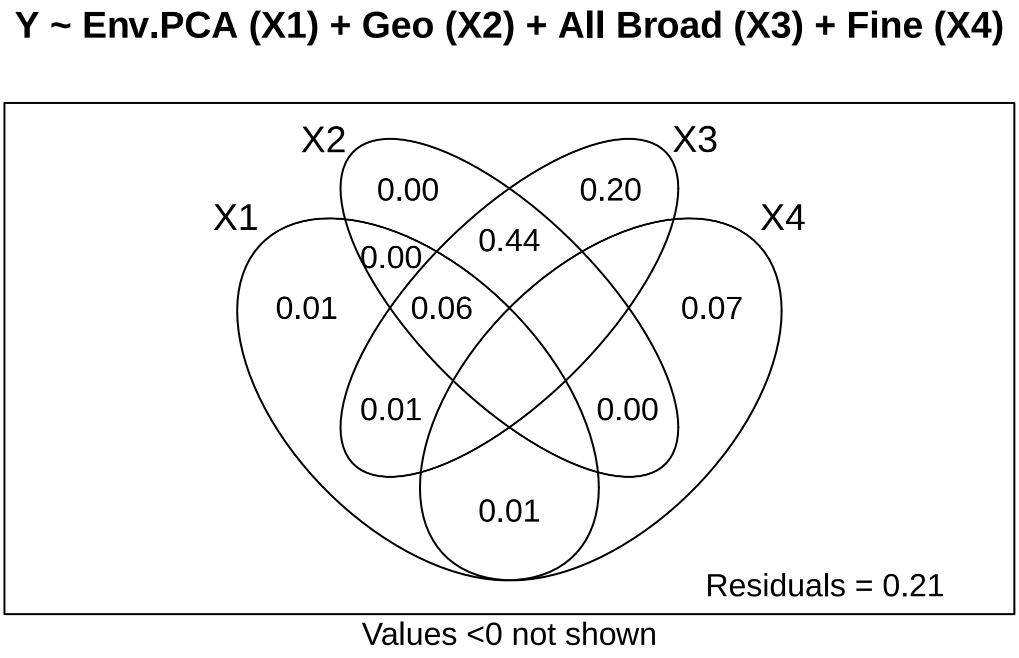

Lets see the RDA axis in the map

```
rda1<-rda(Y.det~., data= as.data.frame(cbind(pca.env, ge0, broad, fine)))
# How much each axes explained?
round(rda1$CCA$eig * 100, 2) / round(sum(rda1$CCA$eig),2)
```

```
##       RDA1       RDA2       RDA3       RDA4       RDA5       RDA6
## 55.0000000 14.1666667  8.3333333  4.3333333  4.3333333  2.8333333
##       RDA7       RDA8       RDA9      RDA10      RDA11      RDA12
##  1.6666667  1.5000000  1.0000000  0.8333333  0.6666667  0.3333333
##      RDA13      RDA14      RDA15      RDA16      RDA17      RDA18
##  0.1666667  0.1666667  0.1666667  0.1666667  0.0000000  0.0000000
##      RDA19      RDA20      RDA21
##  0.0000000  0.0000000  0.0000000
```

```
ax.rda<-scores(rda1, choices=c(1,2), display='lc', scaling=1)

par(mfrow=c(2,1))
plotmap()
    s.value(xy, ax.rda[,1],csize=0.4, pch=22,
        csub=1,xlim=c(-48.5,-44.5),
        ylim=c(-25.5,-23), clegend=1, add.plot=T)
legend("topleft",legend="RDA axis 1", bty='n', cex=2 )
plotmap()
    s.value(xy, ax.rda[,2],csize=0.4, pch=22,
```

```
        csub=2,xlim=c(-48.5,-44.5),
        ylim=c(-25.5,-23), clegend=1, add.plot=T)
legend("topleft",legend="RDA axis 2", bty='n', cex=2)
```

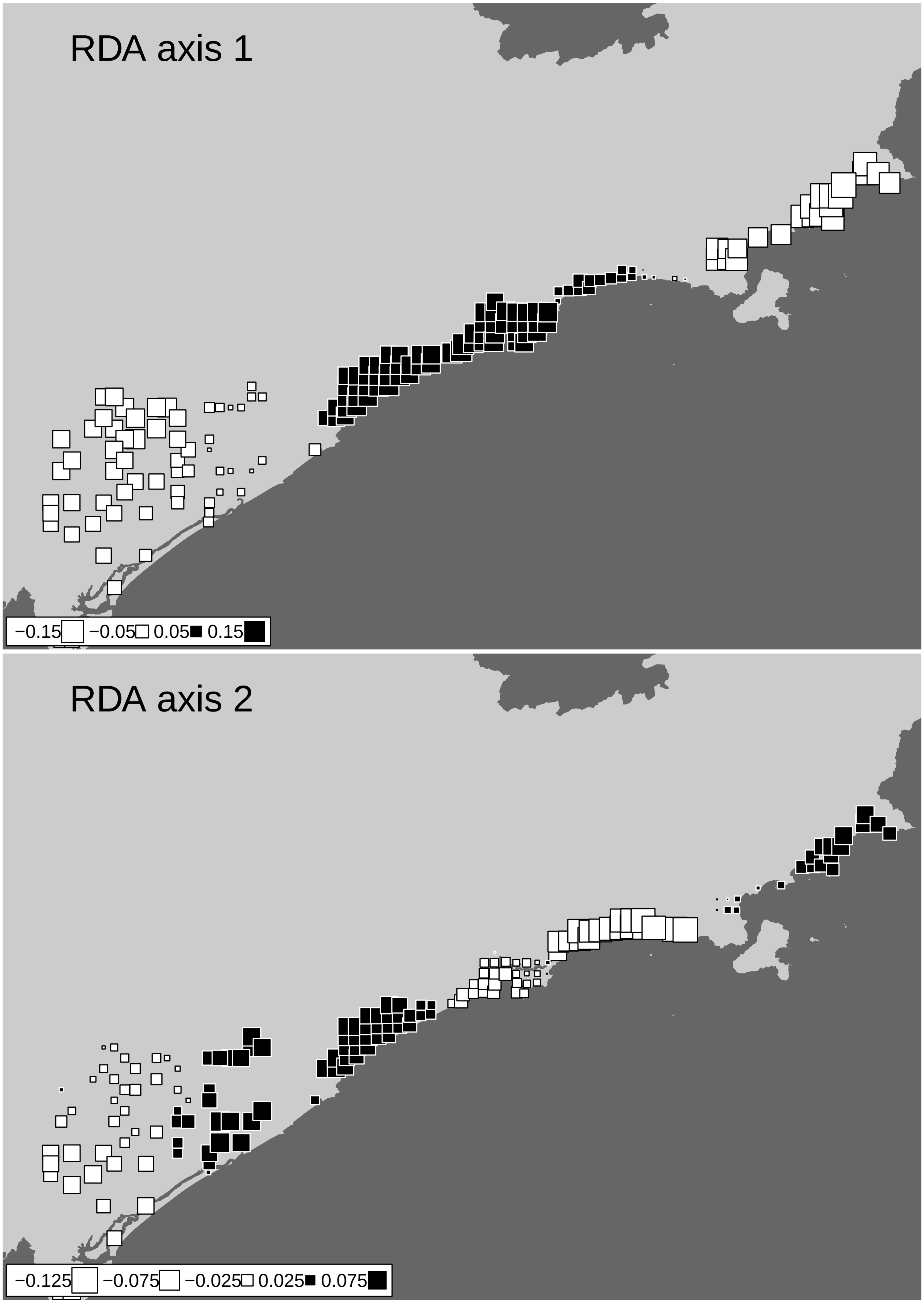
RDA axis 1
−0.15
−0.05
0.05
0.15
RDA axis 2
−0.125
−0.075
−0.025
0.025
0.075

Testing significance of testable individual fractions

```r
#fraction [a] = purely environmental b
anova.cca(rda(Y.det, env, cbind(broad, fine, ge0)), model='direct')
```

```
## Permutation test for rda under direct model
## Permutation: free
## Number of permutations: 999
##
## Model: rda(X = Y.det, Y = env, Z = cbind(broad, fine, ge0))
##           Df  Variance      F Pr(>F)
## Model      4 0.0029552 6.2436  0.001 ***
## Residual 140 0.0165661
## ---
## Signif. codes:  0 '***' 0.001 '**' 0.01 '*' 0.05 '.' 0.1 ' ' 1
```

```r
# fraction [b] = purely Geomorphology
anova.cca(rda(Y.det, ge0, cbind(env, fine, broad)))
```

```
## Permutation test for rda under reduced model
## Permutation: free
## Number of permutations: 999
##
## Model: rda(X = Y.det, Y = ge0, Z = cbind(env, fine, broad))
##           Df Variance      F Pr(>F)
## Model      3 0.018696 52.666  0.001 ***
## Residual 140 0.016566
## ---
## Signif. codes:  0 '***' 0.001 '**' 0.01 '*' 0.05 '.' 0.1 ' ' 1
```

```r
# fraction [c] = purely spatial in fine scale
anova.cca(rda(Y.det, fine, cbind(env, broad, ge0)))
```

```
## Permutation test for rda under reduced model
## Permutation: free
## Number of permutations: 999
##
## Model: rda(X = Y.det, Y = fine, Z = cbind(env, broad, ge0))
##           Df Variance      F Pr(>F)
## Model     10 0.005206 4.3996  0.001 ***
## Residual 140 0.016566
## ---
## Signif. codes:  0 '***' 0.001 '**' 0.01 '*' 0.05 '.' 0.1 ' ' 1
```

```r
# fraction [d] = purely spatial in broad scale
anova.cca(rda(Y.det, broad, cbind(ge0,fine, env)))
```

```
## Permutation test for rda under reduced model
## Permutation: free
## Number of permutations: 999
##
```

```
## Model: rda(X = Y.det, Y = broad, Z = cbind(ge0, fine, env))
##           Df  Variance      F Pr(>F)
## Model      6 0.0088525 12.469  0.001 ***
## Residual 140 0.0165661
## ---
## Signif. codes:  0 '***' 0.001 '**' 0.01 '*' 0.05 '.' 0.1 ' ' 1
```

Testing the neutral dynamic assumptions in the spatial structure of beta diversity (Diniz-Filho et al. 2012)

Preparing some more data to run analysis

```
require (ncf)

Y.hel<-Y
spatial<- cbind(broad,fine) # Complete spatial model

#Defining distance classes

inc<-20
for (i in 1:ncol(Y.hel)){
  xx<-correlog(x=xy.km[,1], y=xy.km[,2], z=Y.hel[,i],
        increment=inc, resamp=1, quiet=TRUE)
  kk<- max(as.numeric(names(xx$n)))
}
```

Here, we'll create the species correlograms (see details in Diniz-Filho et al. 2012) and R and M matrices

```
Correlog<- matrix(NA, nrow=ncol(Y), ncol=kk)

for (i in 1:ncol(Y.hel)){
  corre<-correlog(x=xy.km[,1], y=xy.km[,2], z=Y.hel[,i],
        increment=inc, resamp=1, quiet=TRUE)
  nn<-  length (corre$correlation)
  Correlog[i,]<-as.matrix(corre$correlation)
  rownames(Correlog)<-colnames(Y.hel)
}
```

Plotting Average of Moran's I

```
desvio<-apply(Correlog,2,sd)
media<-colMeans(Correlog)
plot(1:kk,media,ylim=c(-1,1),type="b",
     pch=16,ylab="Average Moran's I",
     xlab="Distance Classes")
lines(1:kk,media+desvio,lty=3)
lines(1:kk,media-desvio,lty=3)
```

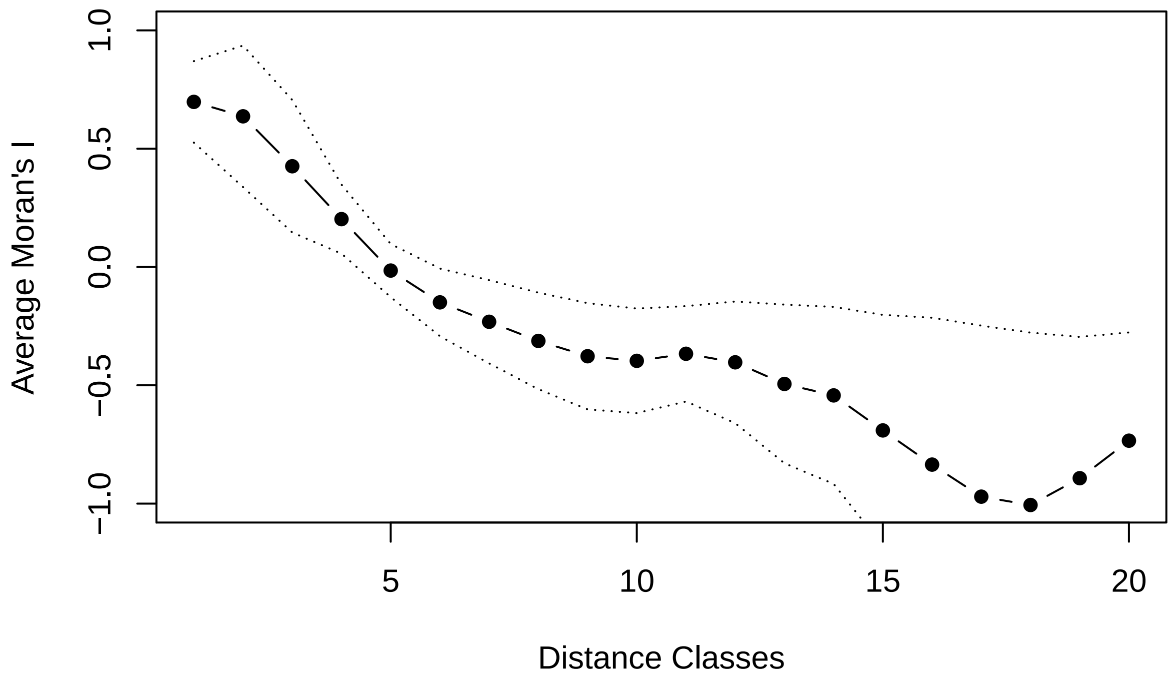

Testing correlation between R and M matrices

```
R<- as.dist(cor(Y.hel))
M<- dist(Correlog, "manhat")
mean(M)
```

```
## [1] 7.120159
```

```
mean(R)
```

```
## [1] 0.04286356
```

```
mantel1<-mantel(xdis=R,ydis=M, permutations=9999)
mantel1
```

```
##
## Mantel statistic based on Pearson's product-moment correlation
##
## Call:
## mantel(xdis = R, ydis = M, permutations = 9999)
##
## Mantel statistic r: -0.5089
##       Significance: 1
##
## Upper quantiles of permutations (null model):
```

```
##     90%     95%  97.5%     99% 
## 0.0487 0.0567 0.0642 0.0705 
## Permutation: free
## Number of permutations: 9999
```

```
plot(M,R, cex=1, pch=20,
     col=rgb(0,0,0,100,maxColorValue=255)
     , ylab= 'R - Incidence Correlations'
     ,xlab='M - Correlogram\'s Manhattan Distance ')
title(main= 'Correlation between R and M matrices')
abline(lm(R~M), col='red', lty=3, lwd=2)
```

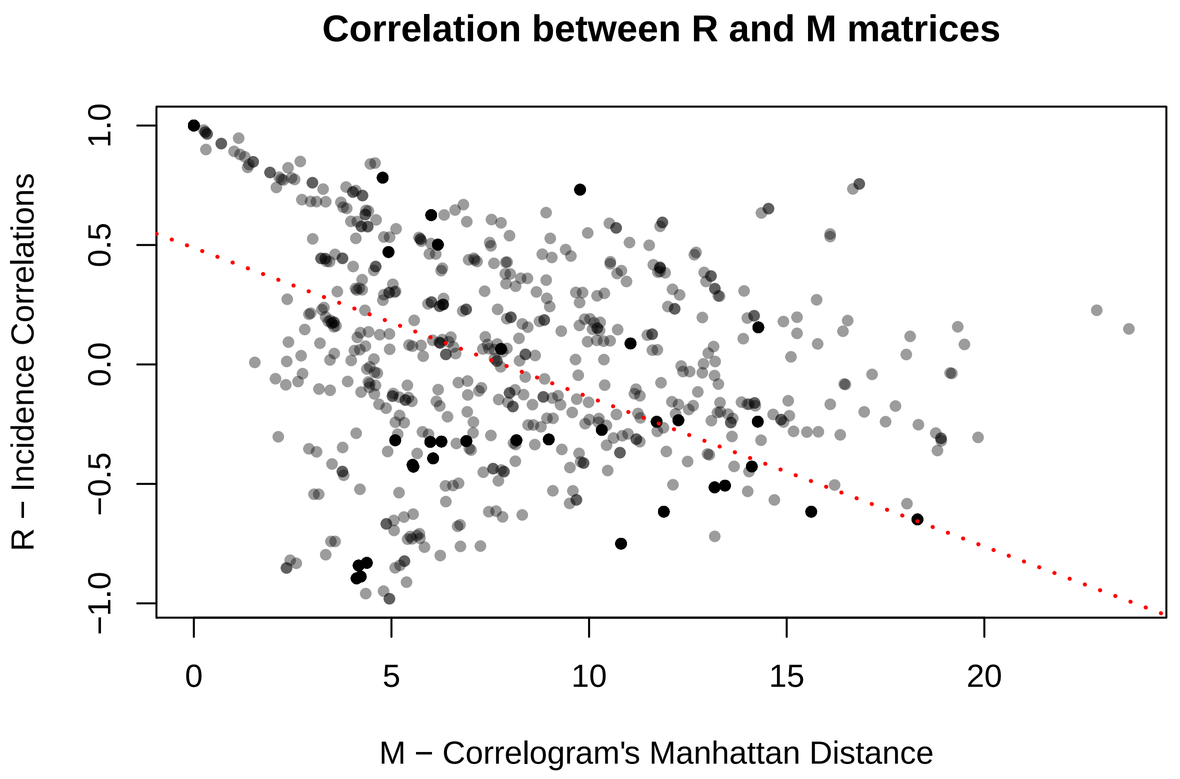

Now, we need follow the same procedure above, but considering the abundance (incidence in our case) predicted only by Spatial model

```
# First we create a full model
full<- rda (Y.hel~., data=as.data.frame(cbind(pca.env,ge0,spatial)))
# Second, we create a model without spatial predictors
env_geo.model<- rda (Y.hel ~.,
        data= as.data.frame (cbind(pca.env,ge0) ))

 # We can test the significance of the model

teste.cca<-anova.cca(full,by="margin",step=99)

# Here, we will create the matrices of predicted values by full model, by model
```

```r
#without spatial predictors, and then subtract them in order to obatain the
#predicted values by spatial model (namely C fraction)

predfull<-predict(full)
predEnv<- predict(env_geo.model)
predSpace<- predfull-predEnv
```

With predicted values by spatial model, we'll create the correlograms, R and M matrices

```r
Correlogc<- matrix(NA, nrow=ncol(Y), ncol=kk)

for (i in 1:ncol(Y)){
  correc<-correlog(x=xy.km[,1], y=xy.km[,2], z=predSpace[,i],
    increment=inc, resamp=1, quiet = TRUE)
  nn<- length (correc$correlation)
  Correlogc[i,]<-as.matrix(correc$correlation)
  rownames(Correlogc)<-colnames(Y)
}
```

Creating R and M matrices:

```r
Rc<- as.dist(cor(predSpace))
Mc<- dist(Correlogc, "manhat")
# Testing correlation btween Rc and Mc
mantel2<-mantel(Mc,Rc, permutations=9999)
mantel2
```

```
##
## Mantel statistic based on Pearson's product-moment correlation
##
## Call:
## mantel(xdis = Mc, ydis = Rc, permutations = 9999)
##
## Mantel statistic r: -0.6952
##       Significance: 1
##
## Upper quantiles of permutations (null model):
##    90%    95%  97.5%    99%
## 0.0731 0.0882 0.1003 0.1133
## Permutation: free
## Number of permutations: 9999
```

```r
desvioc<-apply(Correlogc,2,sd)
mediac<-colMeans(Correlogc)

plot(1:kk,mediac,ylim=c(-1,1),type="b",
         pch=16,ylab="Moran's I",xlab="Distance class")
lines(1:kk,mediac+desvioc,lty=3)
lines(1:kk,mediac-desvioc,lty=3)
```

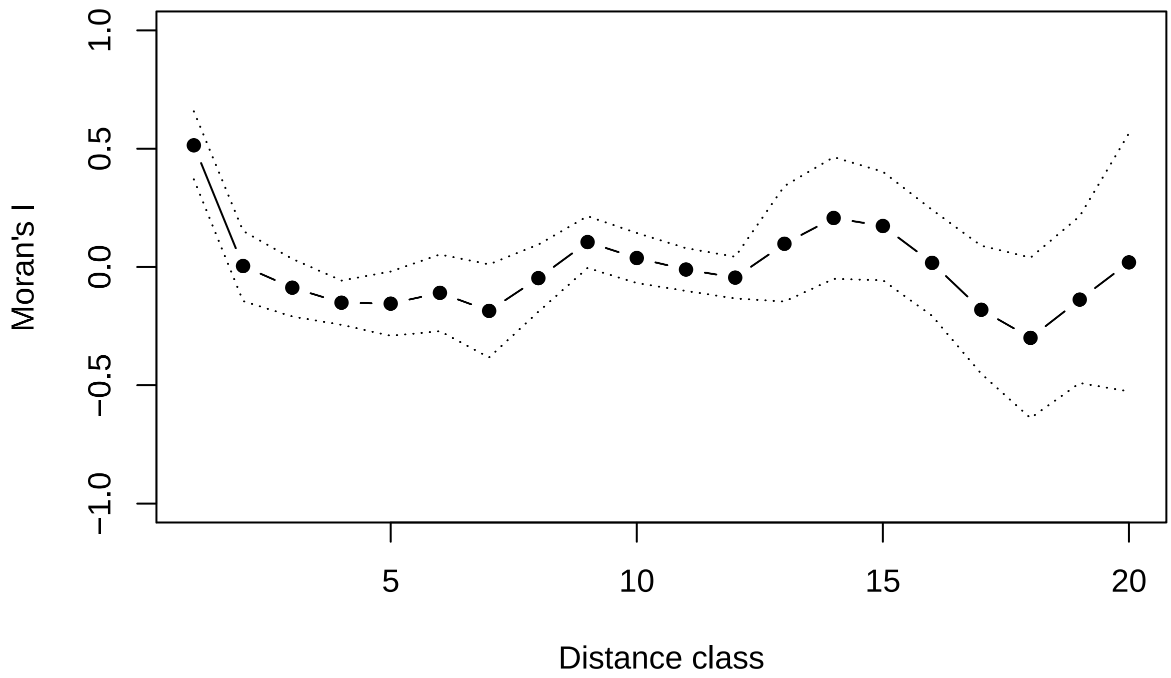


```r
mean(Mc)
```

```
## [1] 4.183645
```

```r
mean (Rc)
```

```
## [1] 0.1151724
```

```r
plot(Mc,Rc, cex=1, pch=20,
     col=rgb(0,0,0,100,maxColorValue=255)
     , ylab= 'Rc - Incidence Correlations'
     ,xlab='Mc - Correlogram\'s Manhattan Distance ')
title(main= 'Correlation between Rc and Mc matrices')
abline(lm(Rc~Mc), col='red', lty=3, lwd=2)
```

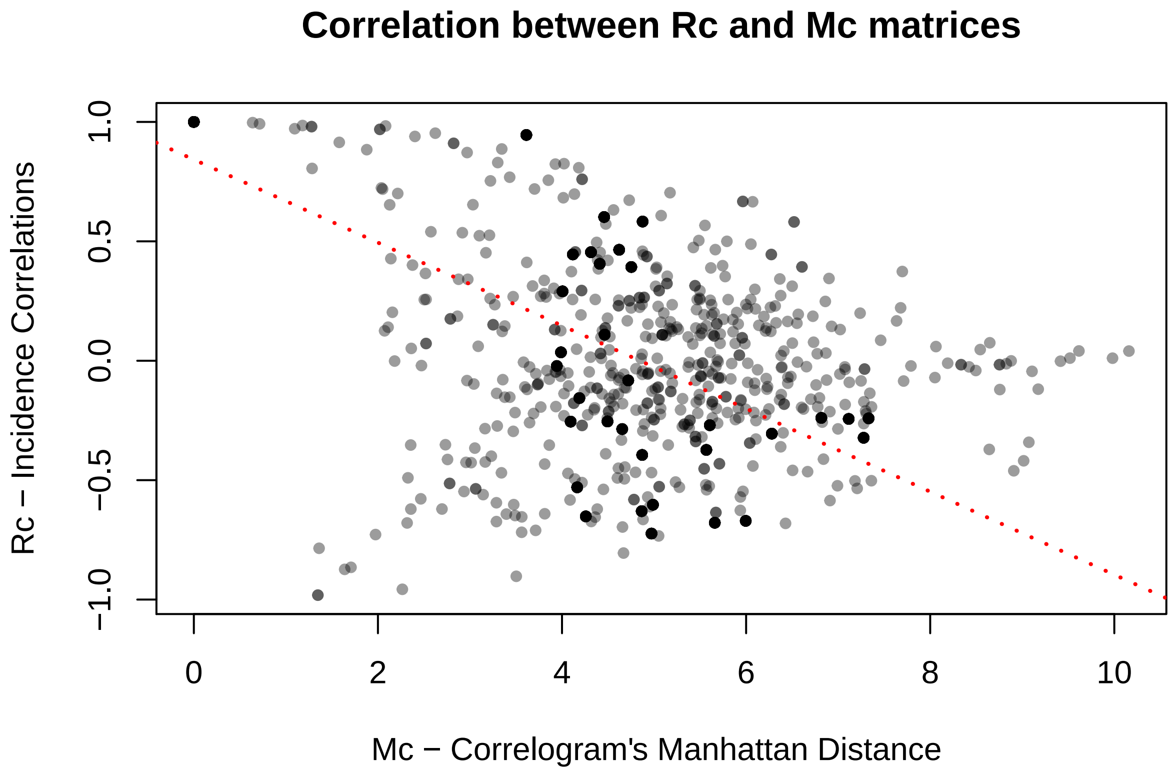

Correlation between Rc and Mc matrices
Rc − Incidence Correlations
1.0
0.5
0.0
−0.5
−1.0
0
2
4
6
8
10
Mc − Correlogram's Manhattan Distance
