# Supplementary material for: Geomorphology Drives Amphibian Beta Diversity in Atlantic Forest Lowlands of Southeastern Brazil
Source: PLoS One. 2016 May 12;11(5):e0153977. doi: 10.1371/journal.pone.0153977 (PMC4865194; doi:10.1371/journal.pone.0153977)
Supplement: S1 Fig — Examples of the Moran Eigenvector Maps (MEMs) classified as broad (i.e., MEM 1 and 2) and fine scales (i.e., MEM 18 and 20), which were used as spatial predictors of the variation of amphibian anuran composition. Squares in the maps represent scores of each site in MEMs. White squares have negative scores and black squares have positive scores. Squares sizes are proportional to score values. These values are also represented in the graphs below each map, allowing to identify similarity in periods (“modulation”) among MEMs. (DOC) [file pone.0153977.s004.doc]

**Supporting Information**

**Geomorphology drives amphibian beta diversity**

**in Atlantic Forest lowlands of southeastern Brazil**

Amom Mendes Luiz, Thiago Augusto Leão-Pires & Ricardo J. Sawaya

**S4 Examples of Moran Eigenvector Maps classified as broad and fine scales**

| 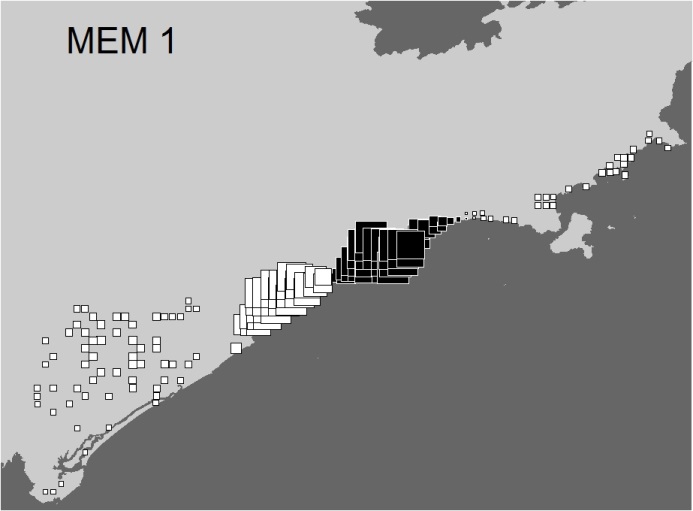 | 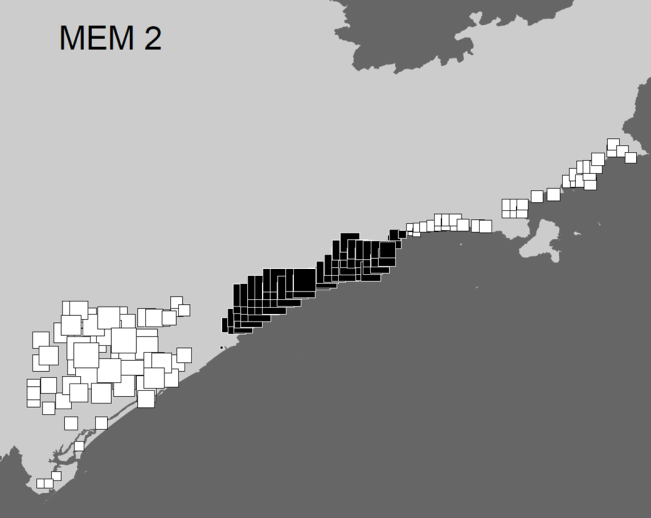 |
| --- | --- |
| 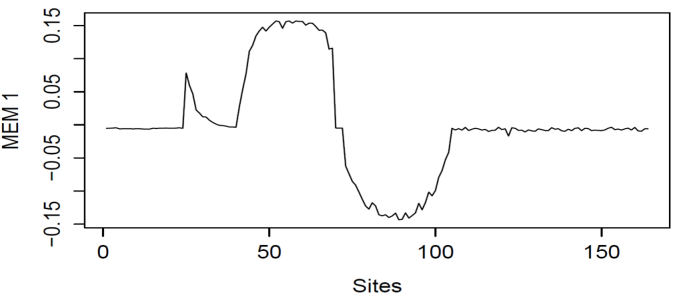 | 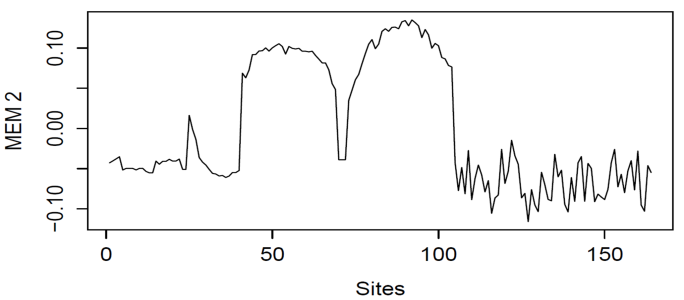 |
| 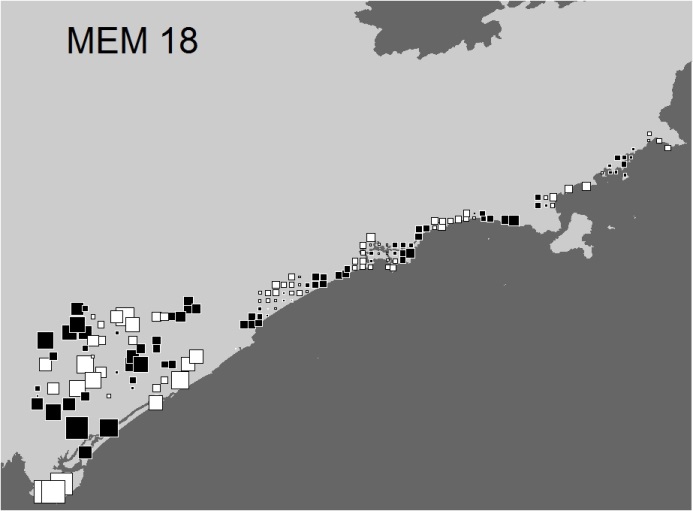 | 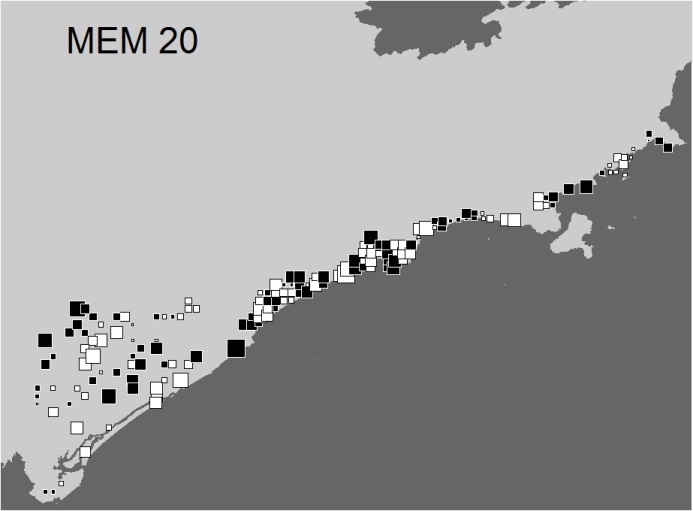 |
| 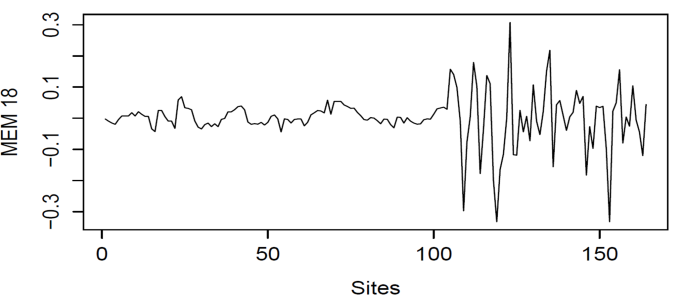 | 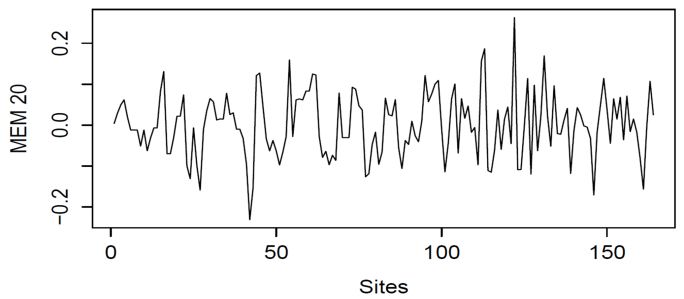 |
